# Chitinase 3 like 1 contributes to the development of pulmonary vascular remodeling in pulmonary hypertension

**DOI:** 10.1172/jci.insight.159578

**Published:** 2022-09-22

**Authors:** Xiuna Sun, Erika Nakajima, Carmelissa Norbrun, Parand Sorkhdini, Alina Xiaoyu Yang, Dongqin Yang, Corey E. Ventetuolo, Julie Braza, Alexander Vang, Jason Aliotta, Debasree Banerjee, Mandy Pereira, Grayson Baird, Qing Lu, Elizabeth O. Harrington, Sharon Rounds, Chun Geun Lee, Hongwei Yao, Gaurav Choudhary, James R. Klinger, Yang Zhou

**Affiliations:** 1 Department of Molecular Microbiology and Immunology and; 2 Department of Pathology and Laboratory Medicine, Brown University, Providence, Rhode Island, USA.; 3 Alpert Medical School of Brown University/Rhode Island Hospital, Providence, Rhode Island, USA.; 4 Providence VA Medical Center, Providence, Rhode Island, USA.; 5 Department of Molecular Biology, Cell Biology, and Biochemistry, Brown University, Providence, Rhode Island, USA.

**Keywords:** Cardiology, Pulmonology, Cardiovascular disease, Endothelial cells, Fibrosis

## Abstract

Chitinase 3 like 1 (CHI3L1) is the prototypic chitinase-like protein mediating inflammation, cell proliferation, and tissue remodeling. Limited data suggest CHI3L1 is elevated in human pulmonary arterial hypertension (PAH) and is associated with disease severity. Despite its importance as a regulator of injury/repair responses, the relationship between CHI3L1 and pulmonary vascular remodeling is not well understood. We hypothesize that CHI3L1 and its signaling pathways contribute to the vascular remodeling responses that occur in pulmonary hypertension (PH). We examined the relationship of plasma CHI3L1 levels and severity of PH in patients with various forms of PH, including group 1 PAH and group 3 PH, and found that circulating levels of serum CHI3L1 were associated with worse hemodynamics and correlated directly with mean pulmonary artery pressure and pulmonary vascular resistance. We also used transgenic mice with constitutive knockout and inducible overexpression of CHI3L1 to examine its role in hypoxia-, monocrotaline-, and bleomycin-induced models of pulmonary vascular disease. In all 3 mouse models of pulmonary vascular disease, pulmonary hypertensive responses were mitigated in CHI3L1-null mice and accentuated in transgenic mice that overexpress CHI3L1. Finally, CHI3L1 alone was sufficient to induce pulmonary arterial smooth muscle cell proliferation, inhibit pulmonary vascular endothelial cell apoptosis, induce the loss of endothelial barrier function, and induce endothelial-mesenchymal transition. These findings demonstrate that CHI3L1 and its receptors play an integral role in pulmonary vascular disease pathobiology and may offer a target for the treatment of PAH and PH associated with fibrotic lung disease.

## Introduction

Pulmonary hypertension (PH), defined most recently as an elevation in mean pulmonary arterial pressure (PAP) over 20 mmHg (1–4), is a common sequela of chronic heart or lung conditions. However, it can also occur as a primary pulmonary vasculopathy in patients without cardiopulmonary disease and is then referred to as pulmonary arterial hypertension (PAH). PAH is rare and may be idiopathic, be associated with drugs or toxins, or occur in patients with systemic conditions such as connective tissue diseases, portal hypertension, and HIV infection (5). PAH also develops in congenital heart disease that causes significant left to right intracardiac shunting, and in patients with a variety of heritable mutations (heritable PAH), most of which are found in the gene for bone morphogenic protein receptor-2.

Common to nearly all forms of PAH is a diffuse, somewhat heterogenous remodeling of the distal pulmonary circulation characterized by the loss of peripheral pulmonary vessels and an obliterative vasculopathy in those that remain (6, 7). Affected vessels exhibit thickening of the vascular intima, media, and adventitia due to endothelial cell proliferation, increased muscularization, and transformation of endothelial cells to mesenchymal cells (endothelial-mesenchymal transition, endo-MT). Perivascular accumulation of monocytes and fibroblasts expressing proinflammatory cytokines and growth factors also appear to play a prominent role. This extensive remodeling leads to marked narrowing or complete obstruction of the vascular lumen, a progressive increase in pulmonary vascular resistance, and eventually right heart failure. Currently available treatment options have been shown to improve functional capacity and delay disease progression but have limited ability to reverse vascular remodeling or lower PAP (8). As a result, 5-year survival in PAH remains around 50%. Thus, there is a need for a better understanding of the mechanisms that drive vascular remodeling in PAH so that more effective therapies can be developed for this devastating disease (9–12).

Pulmonary vascular remodeling and PH are also common in interstitial lung diseases such as idiopathic pulmonary fibrosis (IPF) (group 3 PH) and are independent predictors of mortality (13). The pathogenesis of PH associated with pulmonary fibrosis is not well understood but is thought to be caused by dysregulated vascular repair mechanisms that lead to abnormal vascular cell growth and impaired angiogenesis (9–12, 14–17).

Chitinases are a family of glycoside hydrolases that act as host defense enzymes because they facilitate degradation of chitin — the primary component of cell walls in fungi and the exoskeletons of dust mites and other insects. Chitinase-like proteins resemble chitinases but lack enzymatic activity. They have been shown to play important immunomodulatory roles in a variety of diseases by inducing production of pro- and antiinflammatory cytokines and chemokines. Chitinase 3 like 1 (CHI3L1) is the prototypic chitinase-like protein (18). Studies from our laboratory and others demonstrated that circulating levels of CHI3L1 are higher in individuals with tissue injury and remodeling, including PH and pulmonary fibrosis (19–22). We also demonstrated that CHI3L1 plays a protective role in tissue damage by ameliorating epithelial cell death and a profibrotic role via its ability to stimulate alternative (M2) macrophage activation, fibroblast proliferation, and matrix deposition. Our studies revealed that CHI3L1 regulates cellular injury and repair responses in various cell types via multiple receptors or co-receptors, including IL-13Rα2 (and its co-receptor TMEM219), Galectin-3 (Gal-3), and CRTH2 (23–27). Interestingly, CHI3L1 mediates different responses via these receptors, with IL-13Rα2/TMEM219 inhibiting apoptosis and CRTH2 mediating fibrosis (25, 27). We have also shown that Gal-3 binds CHI3L1 and IL-13Rα2 and that it modulates CHI3L1 effector functions in inflammation, cellular apoptosis, and tissue remodeling by competing with TMEM219 for binding to IL-13Rα2 (26). Despite its importance as a regulator of injury/repair and inflammatory responses, its role in mediating pulmonary vascular remodeling in PAH and PH associated with pulmonary fibrosis is not known.

In the present study, we hypothesize that CHI3L1 and its signaling pathways contribute to the vascular remodeling responses that occur in PH. We found that circulating levels of serum CHI3L1 were associated with worse hemodynamics in patients with variable subtypes of PH. Furthermore, overexpression of CHI3L1 worsened PH in hypoxia-, monocrotaline-, and bleomycin-induced models of PH, whereas suppression of its expression attenuated PH. In vitro, CHI3L1 directly stimulated pulmonary arterial smooth muscle cell proliferation and was a potent inhibitor of endothelial cell death responses via various cell surface CHI3L1 receptors. In addition, CHI3L1 synergized with TGF-β and hypoxia to promote the loss of endothelial barrier function and endo-MT. Together, these studies strongly suggest that CHI3L1 and its receptors play an important role in mediating pulmonary vascular remodeling and PH.

## Results

### Circulating levels of serum CHI3L1 are associated with worse hemodynamics in patients with PH.

**Figure 1:**
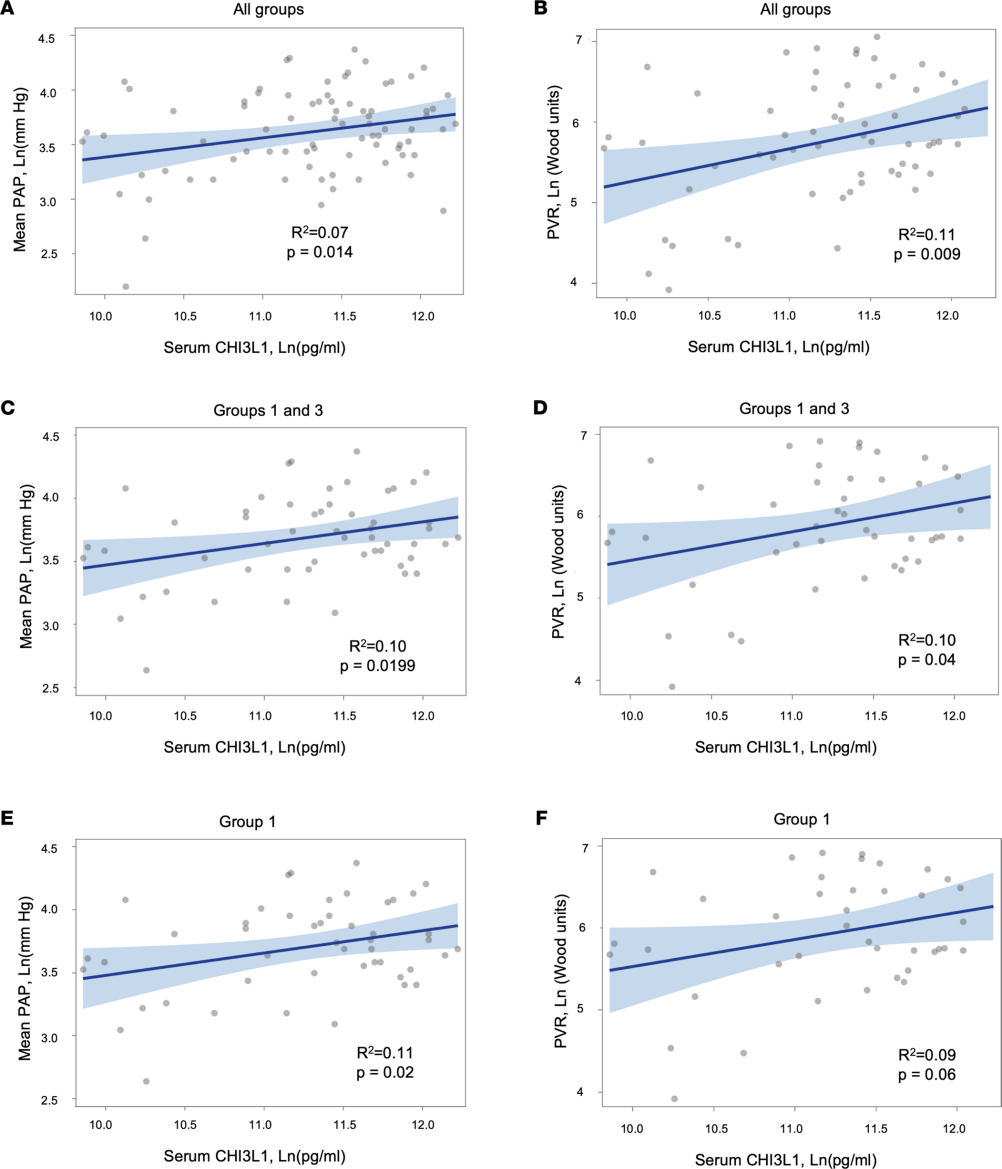
Association between Ln(CHI3L1) levels and hemodynamics in all patients with available hemodynamics. A generalized linear mixed effect model was used to examine associations between Ln(CHI3L1) levels and mPAP and PVR. (**A** and **B**) All patients. (**C** and **D**) Combined patients with group 1 PAH and group 3 PH. (**E** and **F**) Group 1 PAH patients alone. Gray circles are individual data points, dark blue line is regression line, and light blue lines represent 95% confidence bands. Parametric data were compared by 2-tailed Student’s *t* test. Nonparametric data were compared using the Mann-Whitney *U* test. PAP, pulmonary artery pressure; PVR, pulmonary vascular resistance.

Serum CHI3L1 levels were measured from 105 patients including diseased controls (no PH; as defined by a cutoff of a mean PAP < 20 mmHg) (3, 4) and World Symposium on Pulmonary Hypertension (WSPH) groups 1 through 5. Eighty-nine (85%) had PH (WSPH groups 1–5), and 16 (15%) were diseased controls (Table 1). Among all patients with hemodynamics performed and irrespective of WSPH group, there was a direct correlation between CHI3L1 levels and mean pulmonary artery pressure (mPAP) (*R*^2^ = 0.075, *P* = 0.014) (Figure 1A) and pulmonary vascular resistance (*R*^2^ = 0.11, *P* = 0.009) (Figure 1B). In group 1 and 3 patients, there was a direct correlation between CHI3L1 levels and mPAP (*R*^2^ = 0.10, *P* = 0.0199) (Figure 1C) and pulmonary vascular resistance (*R*^2^ = 0.10, *P* = 0.04) (Figure 1D). In group 1 PAH patients alone, there was a direct correlation between CHI3L1 levels and mPAP (*R*^2^ = 0.11, *P* = 0.025) (Figure 1E) and pulmonary vascular resistance (*R*^2^ = 0.09, *P* = 0.06) (Figure 1F).

### CHI3L1 plays a critical role in vascular remodeling responses in a hypoxia-induced PH model.

**Figure 2:**
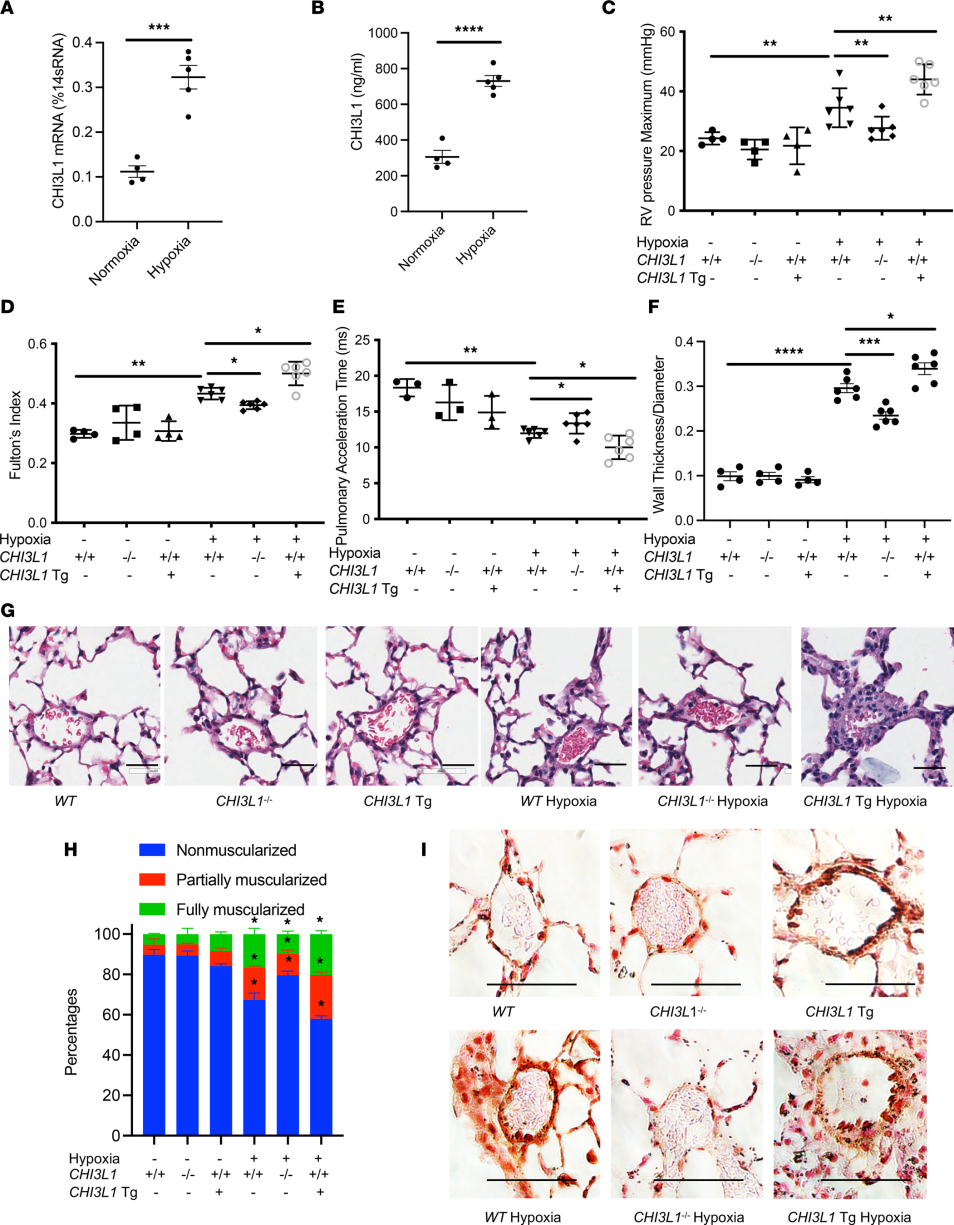
CHI3L1 plays a critical role in vascular remodeling responses in a hypoxia-induced PH model. *WT* (+/+), *CHI3L1*-null mice (-/-), and *CHI3L1*-transgenic overexpression mice (Tg+) were subjected to 6 weeks of normoxia or hypoxia (10% oxygen). (**A**) Whole lung RNA was extracted and *CHI3L1* mRNA levels were measured by real-time PCR (RT-PCR). (**B**) CHI3L1 protein levels were measured in the BAL fluid by ELISA. (**C**) RVSP was measured by right heart catheterization. (**D**) Fulton’s index was calculated based on the dry weight of right ventricle and left ventricle plus the septum. (**E**) Echocardiography was used to calculate the PAT. (**F**) Aperio digital pathology slide scanner was used to assess the medial remodeling of pulmonary arteries. The thickness of the medial layer was expressed as a fraction of the external diameter of the pulmonary artery. At least 40 vessels were examined in each group of normoxic mice, and at least 60 vessels were examined in each group of hypoxic mice. (**G**) H&E staining of the lungs from *WT* mice, *CHI3L1*-null mice, and *CHI3L1*-Tg mice exposed to normoxia or hypoxia. Scale bar, 50 μm. (**H**) Vessel wall muscularization of distal pulmonary arteriole (<100 μm in diameter) was quantified based on immunostaining of α-SMA. (**I**) α-SMA staining of the lungs from *WT* mice, *CHI3L1*-null mice, and *CHI3L1*-Tg mice exposed to normoxia or hypoxia. Scale bar, 50 μm. Values are mean ± SEM with 4–6 mice in each group. Groups were compared by ANOVA with Bonferroni’s posttest; follow-up comparisons between groups were conducted using a 2-tailed Student’s *t* test. *P *≤* 0.05, **P *≤* 0.01, ***P *≤* 0.001, ****P *≤* 0.0001. Images are representatives of 4–6 mice in each group.

To establish the chronic hypoxia PH model, mice were exposed to 4 weeks of hypoxia (10% O_2_) or normoxia, as a control treatment. *CHI3L1* whole lung mRNA transcript and bronchoalveolar lavage (BAL) protein levels were both higher in mice exposed to hypoxia (Figure 2, A and B). As shown in Figure 2C, mice exposed to hypoxia had increased right ventricular (RV) systolic pressure (RVSP), which was determined by invasive hemodynamic measurement. Hypoxic mice also developed RV hypertrophy, which was indicated by a marked increase in the ratio of right ventricle weight to left ventricle plus septum weight (Fulton’s index) (Figure 2D). PAP as assessed by pulmonary artery acceleration time (PAT) was significantly increased in mice exposed to hypoxia (Figure 2E). Consistent with these findings, histological evaluation demonstrated an increase in vascular wall thickening (Figure 2, F and G, Supplemental Figure 1A).

To evaluate the role of CHI3L1 in pulmonary vascular remodeling responses in the hypoxia model, we compared these parameters in wild-type (*WT*) mice versus CHI3L1-null mice (*CHI3L1*^–/–^) and mice with inducible, lung-specific transgenic overexpression of CHI3L1 (*CHI3L1*-Tg). We found that hypoxia-induced increases in RV hypertrophy, vascular wall thickness, and PAP measured by RVSP were significantly diminished in *CHI3L1*^–/–^ mice and significantly exacerbated in *CHI3L1*-Tg mice (Figure 2, A–G). Consistently, hypoxia-induced decreased PAT was reversed in *CHI3L1*^–/–^ mice and further decreased in *CHI3L1*-Tg mice (Figure 2E). Immunostaining for α–smooth muscle actin (α-SMA) showed that muscularization of distal pulmonary arterioles (<100 μm in diameter) in hypoxia-exposed mice was diminished in *CHI3L1*^–/–^ mice and further exacerbated in *CHI3L1*-Tg mice (Figure 2, H and I, and Supplemental Figure 1B). These findings demonstrate that CHI3L1 plays a key role in modulating PH in mice exposed to chronic hypoxia.

### CHI3L1 plays a critical role in vascular remodeling responses in monocrotaline-induced PH.

**Figure 3:**
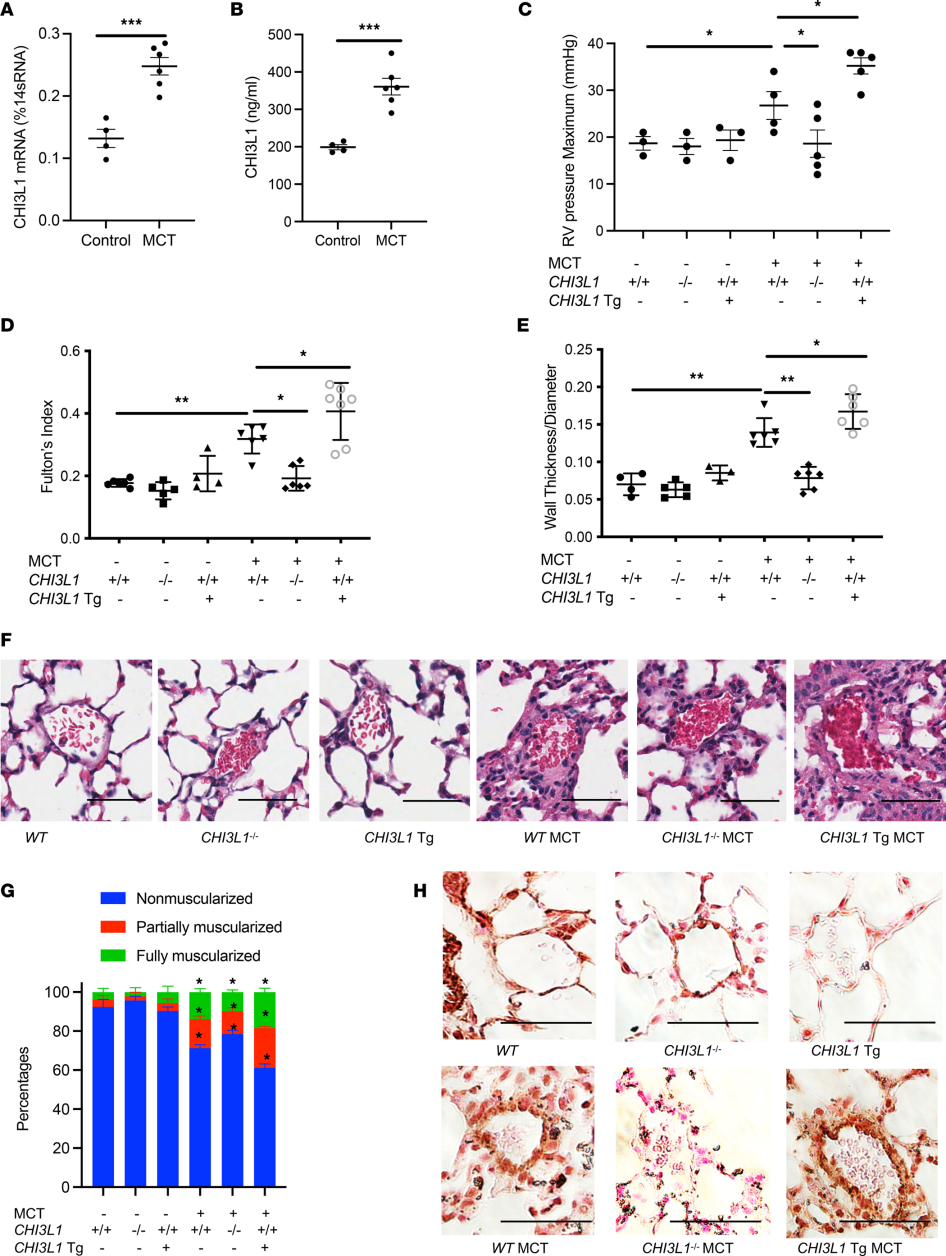
CHI3L1 plays a critical role in vascular remodeling responses in an MCT-induced PH model. *WT* (+/+), *CHI3L1*-null mice (-/-), and *CHI3L1*-transgenic overexpression mice (Tg+) were challenged with MCT (600 mg/kg weekly for 4 weeks) or vehicle and sacrificed 1 week after the last MCT injection. (**A**) Whole lung RNA was extracted and *CHI3L1* mRNA levels were measured by RT-PCR. (**B**) CHI3L1 protein levels were measured in the BAL fluid by ELISA. (**C**) RVSP was measured by right heart catheterization. (**D**) Fulton’s index was calculated based on the dry weight of right ventricle and left ventricle plus the septum. (**E**) Aperio digital pathology slide scanner was used to assess the medial remodeling of pulmonary arteries. The thickness of the medial layer was expressed as a fraction of the external diameter of the pulmonary artery. (**F**) H&E staining of the lungs from *WT* mice, *CHI3L1*-null mice, and *CHI3L1*-Tg mice challenged with MCT (10 mg/kg) or vehicle controls. (**G**) Vessel wall muscularization of distal pulmonary arterioles (<100 μm in diameter) was quantified based on immunostaining of α-SMA. (**H**) α-SMA staining of the lungs from *WT* mice, *CHI3L1*-null mice, and *CHI3L1*-Tg mice challenged with MCT (10 mg/kg) or vehicle controls. Scale bar, 50 μm. Values are mean ± SEM with 4–7 mice in each group. Groups were compared by ANOVA with Bonferroni’s posttest; follow-up comparisons between groups were conducted using a 2-tailed Student’s *t* test. *P *≤* 0.05, **P *≤* 0.01, ***P *≤* 0.001. Images are representatives of 4–7 mice in each group.

The monocrotaline (MCT) mouse model mimics several aspects of human PAH, including endothelial remodeling, smooth muscle proliferation, and RV dysfunction (28–30). To evaluate the role of CHI3L1 in pulmonary vascular remodeling responses in alternative models of PH, we treated mice with MCT (600 mg/kg weekly for 4 weeks) and evaluated RV hypertrophy and vascular remodeling. Mice treated with MCT had increased CHI3L1 whole lung mRNA transcript and BAL protein levels (Figure 3, A and B) compared with controls and developed severe PH as evidenced by significant increases in Fulton’s index and pulmonary vascular wall thickness (Figure 3, C and D, and Supplemental Figure 2A). MCT-induced increases in RVSP and Fulton’s index were completely absent in mice with null mutations of CHI3L1 and significantly augmented in mice with CHI3L1 overexpression (Figure 3, C and D). Imaging analysis further confirmed a decrease in MCT-induced medial wall thickening of small pulmonary arteries in the *CHI3L1*^–/–^ mice and increased wall thickening in *CHI3L1*-Tg mice compared with WT mice (Figure 3, E and F). Immunostaining for α-SMA showed that muscularization of distal pulmonary arterioles (<100 μm in diameter) in MCT-challenged mice was diminished in *CHI3L1*^–/–^ mice and further exacerbated in *CHI3L1*-Tg mice (Figure 3, G and H, and Supplemental Figure 2B). As in the chronic hypoxia model, these findings demonstrate that CHI3L1 plays a key role in modulating RV hypertrophy and pulmonary vascular remodeling in the MCT model of PH.

### CHI3L1 plays a critical role in vascular remodeling responses in a bleomycin-induced pulmonary fibrosis model.

**Figure 4:**
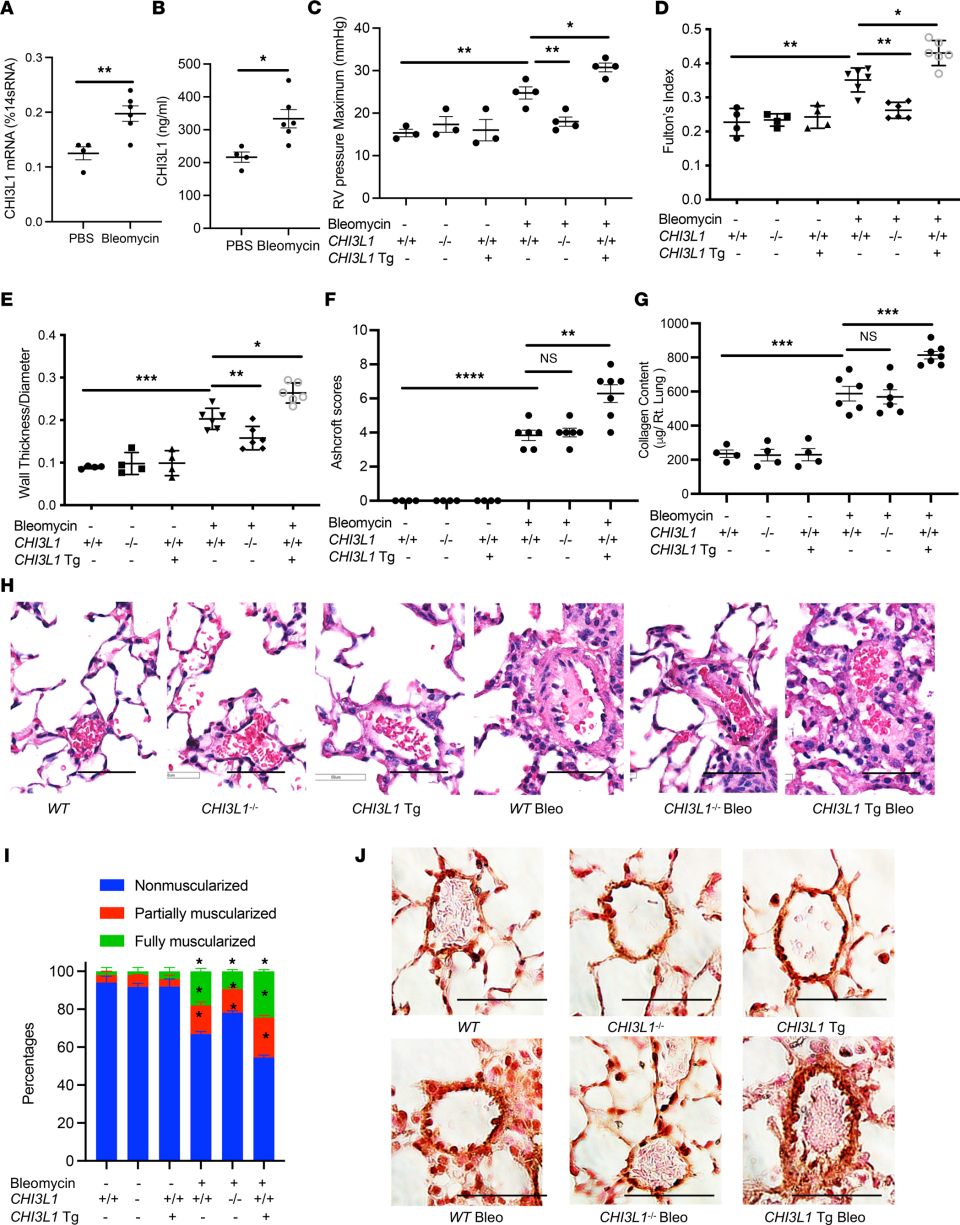
CHI3L1 plays a critical role in vascular remodeling responses in a bleomycin-induced pulmonary fibrosis model. *WT* (+/+), *CHI3L1*-null mice (-/-), and *CHI3L1*-transgenic overexpression mice (Tg+) were subjected to intratracheal PBS or bleomycin administration. Mice were sacrificed at day 14 after bleomycin challenge. (**A**) Whole lung RNA was extracted and *CHI3L1* mRNA levels were measured by RT-PCR. (**B**) CHI3L1 protein levels were measured in the BAL fluid by ELISA. (**C**) RVSP was measured by right heart catheterization. (**D**) Fulton’s index was calculated based on the dry weight of right ventricle and left ventricle plus the septum. (**E**) Aperio digital pathology slide scanner was used to assess the medial remodeling of pulmonary arteries. The thickness of the medial layer was expressed as a fraction of the external diameter of the pulmonary artery. (**F**) Ashcroft histology scores were assessed to evaluate levels of lung fibrosis. (**G**) Collagen deposition in the lung was assessed by Sircol assay. (**H**) H&E staining of the lungs from *WT* mice, *CHI3L1*-null mice, and *CHI3L1*-Tg mice challenged with bleomycin. (**I**) Vessel wall muscularization of distal pulmonary arterioles (<100 μm in diameter) was quantified based on immunostaining of α-SMA. (**J**) α-SMA staining of the lungs from *WT* mice, *CHI3L1*-null mice, and *CHI3L1*-Tg mice challenged with bleomycin. Scale bar, 50 μm. Values are mean ± SEM with 4–6 mice in each group. Groups were compared by ANOVA with Bonferroni’s posttest; follow-up comparisons between groups were conducted using a 2-tailed Student’s *t* test. *P *≤* 0.05, **P *≤* 0.01, ***P *≤* 0.001. Images are representatives of 4 to 6 mice in each group.

Intratracheal bleomycin instillation is frequently used to induce lung fibrosis followed by acute lung injury (31–35). While systemic bleomycin is known to induce vascular injury, intratracheally bleomycin-treated mice develop key pathophysiological features of PH associated with pulmonary fibrosis, including pulmonary vascular remodeling and increased RV pressure (36–48). To evaluate the role of CHI3L1 in pulmonary vascular remodeling responses associated with pulmonary fibrosis, we treated mice with a single dose of intratracheal bleomycin (1.25 U/kg) and evaluated RV hypertrophy and vascular remodeling responses. Mice treated with bleomycin had increased CHI3L1 whole lung mRNA transcript and BAL protein levels (Figure 4, A and B) and developed significant PH as evidenced by increased RVSP, the development of RV hypertrophy, and increased pulmonary vessel wall thickening (Figure 4, C–E, and Supplemental Figure 3A). Similar to our findings in the chronic hypoxia and MCT-induced models of PH, we found that *CHI3L1*^–/–^ mice that were treated with bleomycin had markedly decreased RVSP, RV hypertrophy, and vascular wall thickening compared with WT mice (Figure 4, C–E and H), whereas RV hypertrophy and vascular remodeling responses were augmented in the *CHI3L1*-Tg mice (Figure 4, C–E). Interestingly, bleomycin-induced fibrotic response and collagen deposition were not reduced in *CHI3L1*^–/–^ mice (Figure 4, F and G). α-SMA immunostaining showed that muscularization of distal pulmonary arterioles (<100 μm in diameter) in bleomycin-challenged mice was diminished in *CHI3L1*^–/–^ mice and further exacerbated in *CHI3L1*-Tg mice (Figure 4, I and J, and Supplemental Figure 3B). These findings demonstrate that CHI3L1 plays a key role in modulating PH in the bleomycin model of pulmonary fibrosis.

### CHI3L1 stimulates vascular smooth muscle cell proliferation.

**Figure 5:**
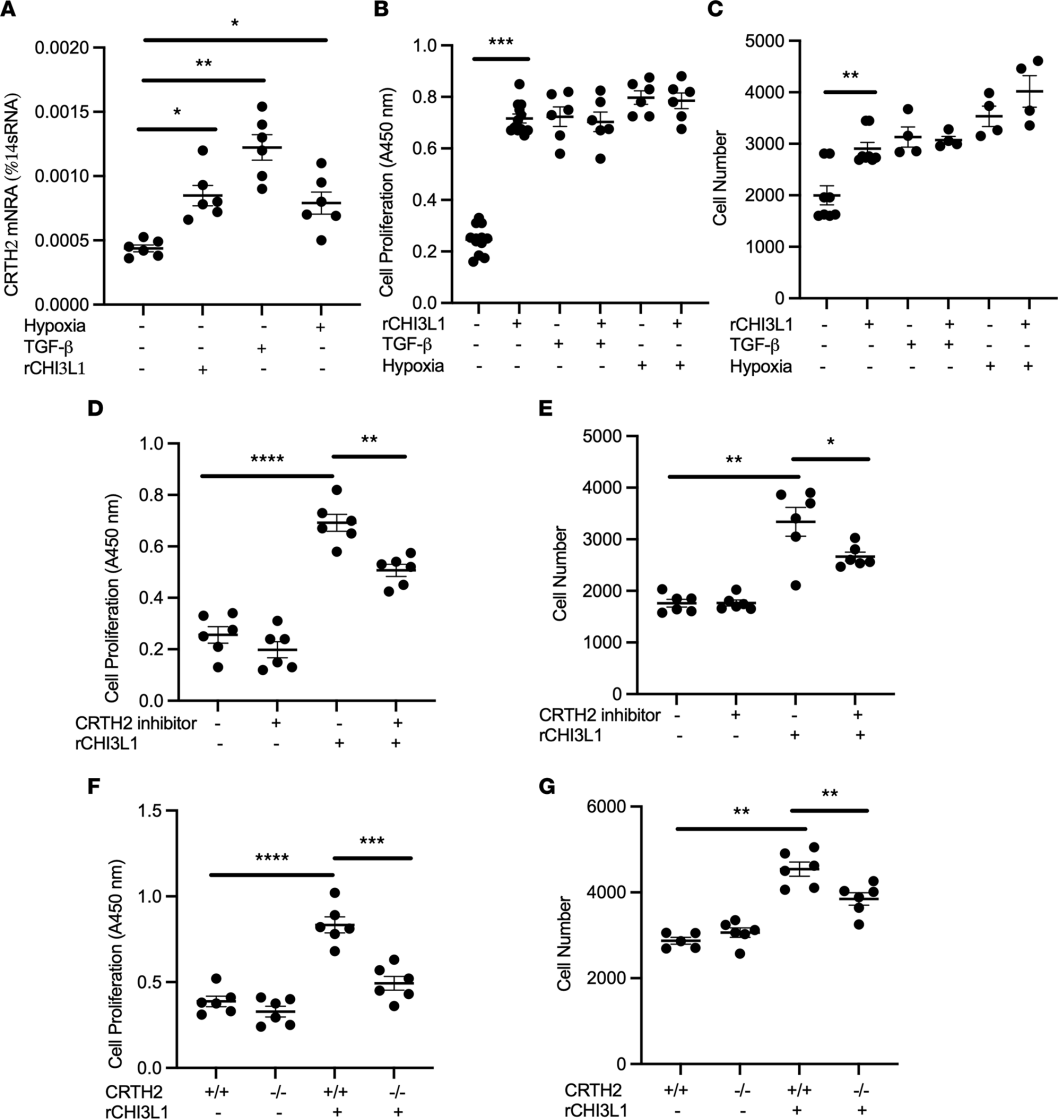
CHI3L1 stimulates smooth muscle cell proliferation through interaction with CRTH2. Human pulmonary arterial smooth muscle cells (HPASMCs, Lonza) were treated with CHI3L1, TGF-β, or hypoxia. (**A**) Transcript levels of CRTH2 were measured in RNA extracts using RT-PCR. rCHI3L1, recombinant CHI3L1. (**B**) HPASMCs were treated with CHI3L1 (500 ng/mL), TGF-β (10 ng/mL), or both for 48 hours. In a separate experiment, cells were treated with CHI3L1 (500 ng/mL), exposed to hypoxia (1% oxygen), or both. Cell proliferation was quantitated by BrdU incorporation ELISA (**B**), and cell number was quantitated by WST-1 assay (**C**). HPASMCs were treated with CHI3L1 with or without CRTH2 inhibitor CAY10471. Cell proliferation was quantitated by BrdU incorporation ELISA (**D**), and cell number was quantitated by WST-1 assay (**E**). DMSO was used as vehicle control. Primary mouse PASMCs were isolated from WT and CRTH2-null mice. Cells were treated with CHI3L1, proliferation was quantitated by BrdU incorporation ELISA (**F**), and cell number was quantitated by WST-1 assay (**G**). Values are mean ± SEM with 6 wells in each group. Each experiment was undertaken at least 3 times. Groups were compared by ANOVA with Bonferroni’s posttest; follow-up comparisons between groups were conducted using a 2-tailed Student’s *t* test. *P *≤* 0.05, **P *≤* 0.01, ***P *≤* 0.001, ****P *≤* 0.0001.

The proliferation of vascular smooth muscle cells (VSMCs) is important in the progression of PH, and effective inhibition of aberrant VSMC proliferation can delay, and even halt, PH progression (49). Our preliminary studies demonstrated that although CHI3L1 was not expressed in human pulmonary artery smooth muscle cells (HPASMCs), we were able to detect the expression of various CHI3L1 receptor systems. Specifically, we demonstrated that CRTH2, a G protein–coupled receptor that CHI3L1 interacts with, was expressed by VSMCs and that both CHI3L1 and the fibrotic stimuli, TGF-β or hypoxia, significantly increased its expression (Figure 5A). Studies were undertaken to examine the role of CRTH2 on VSMCs in response to CHI3L1 stimulation. HPASMCs were stimulated with CHI3L1 (500 ng/mL), and cell proliferation was assessed by BrdU incorporation ELISA (Figure 5B) and WST-1 Cell Proliferation Assay (Figure 5C). The additive or synergistic effect of rCHI3L1 in growth factor–stimulated and hypoxia-induced cell proliferation was also evaluated. We found that, as expected, TGF-β and hypoxia were both strong stimuli of VSMC proliferation. Although CHI3L1 did not seem to synergize with either to further stimulate cell proliferation, CHI3L1 itself was able to drive the proliferative effect of HPASMCs (Figure 5, B and C). The role of CRTH2 as a CHI3L1 receptor on smooth muscle cells was further validated by treating cells with a small molecule inhibitor of CRTH2 or using primary mouse pulmonary smooth muscle cells isolated from *CRTH2*-null mice. We found that CHI3L1-induced cell proliferation was significantly diminished in cells that were treated with CRTH2 inhibitor (Figure 5, D and E) or cells lacking CRTH2 expression (Figure 5, F and G). These results demonstrate that CHI3L1 stimulates VSMC proliferation at least partially, via the CRTH2 receptor.

### CHI3L1 binds with IL-13Rα2 and TMEM219 and inhibits pulmonary arterial endothelial cell death.

**Figure 6:**
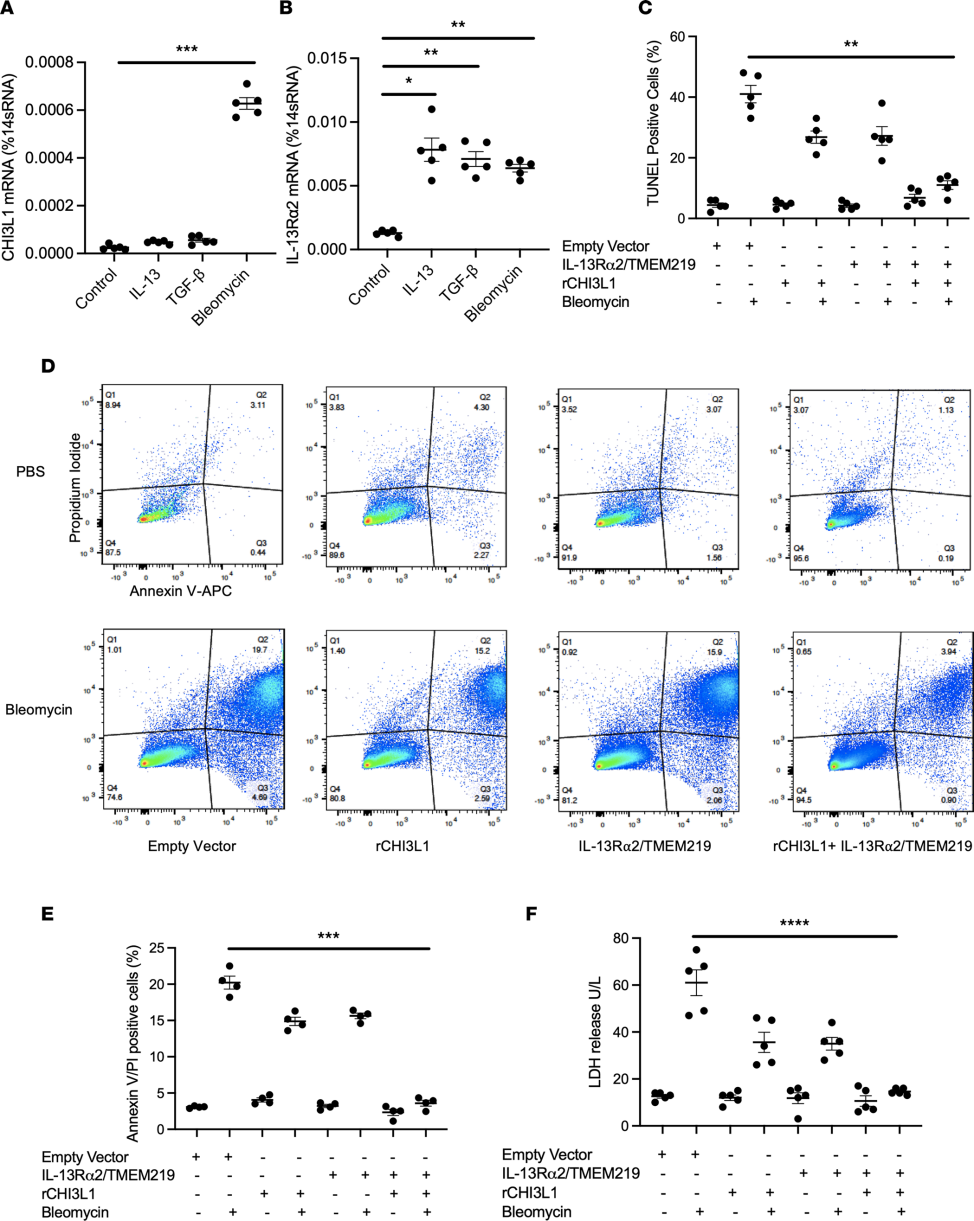
CHI3L1 binds with IL-13Rα2 and TMEM219 and inhibits pulmonary arterial endothelial cell death. (**A**) Human pulmonary arterial endothelial cells (HPAECs, Lonza) were treated with IL-13, TGF-β, or bleomycin for 16 hours. Transcript levels of (**A**) CHI3L1 and (**B**) IL-13Rα2 were measured in RNA extracts using RT-PCR. (**C**) HPAECs were transfected with empty vector or IL-13Rα and TMEM219 constructs simultaneously and treated with recombinant CHI3L1 with or without bleomycin stimulation in vitro. TUNEL staining was performed and TUNEL-positive cells were counted. Values are mean ± SEM with a minimum of 4 wells in each group. (**D**) HPAECs were treated with the same conditions as previously, then stained with annexin V–APC for 15 minutes. Propidium iodide (PI) was added 5 minutes before FACS analysis. (**E**) Quantifications of annexin V/PI–positive cells. (**F**) Lactate dehydrogenase (LDH) assay was performed to examine the release of LDH into cell culture medium. Each experiment was undertaken at least 3 times. Groups were compared by ANOVA with Bonferroni’s posttest; follow-up comparisons between groups were conducted using a 2-tailed Student’s *t* test. ***P *≤* 0.001, **P *≤* 0.01, *P *≤* 0.05 compared with controls.

Endothelial cell apoptosis has been recognized as a key feature of pulmonary vascular remodeling in PH. Numerous studies have shown that endothelial cell apoptosis initiates vascular remodeling responses in various models of PH, and increased proliferation of apoptosis-resistant endothelial cells has been identified in plexiform lesions of patients with PAH. To characterize the expression profiles of CHI3L1 and its receptor systems in endothelial cells, we treated human pulmonary arterial endothelial cells (HPAECs) with fibrotic stimuli, including IL-13 (20 ng/mL), TGF-β (10 ng/mL), and bleomycin (200 μg/mL). Our results demonstrated that CHI3L1 and IL-13Rα2 were expressed by endothelial cells and that fibrotic stimuli increased the expression of CHI3L1 and IL-13Rα2 (Figure 6, A and B). Our previous studies demonstrated that CHI3L1/IL-13Rα2 interaction requires a co-receptor, TMEM219 (23). We next challenged HPAECs with bleomycin to induce cell death and determined if CHI3L1/IL-13Rα2/TMEM219 signaling was able to inhibit the cell death response in endothelial cells by TUNEL staining (Figure 6C), annexin V–propidium iodide (PI) staining (Figure 6, D and E), and measuring lactate dehydrogenase (LDH) release in cell culture medium (Figure 6F). In these studies, treatment with rCHI3L1 or overexpression of IL-13Rα2/TMEM219 partially reduced bleomycin-induced apoptosis, and the combination of rCHI3L1 treatment and IL-13Rα2/TMEM219 overexpression blocked it completely (Figure 6, C–F). Thus, these studies identify IL-13Rα2/TMEM219 as a signaling receptor for CHI3L1, mediating its antiapoptotic effects in endothelial cells.

### CHI3L1 synergizes with TGF-β and hypoxia to promote endothelial permeability and endo-MT transition.

**Figure 7:**
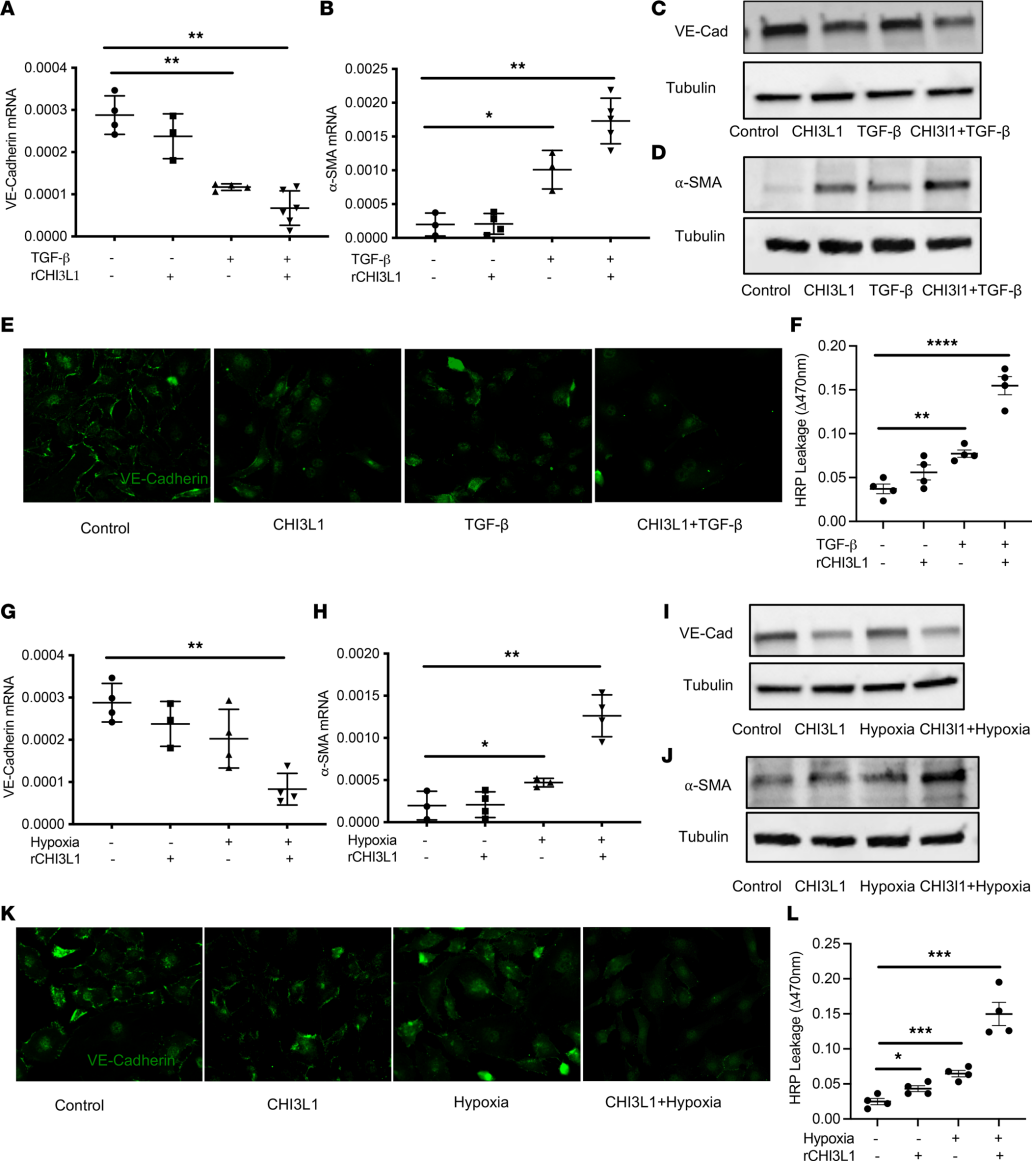
CHI3L1 synergizes with TGF-β or hypoxia to promote endothelial permeability and endo-MT. BPAECs were treated with CHI3L1 (500 ng/mL), TGF-β (10 ng/mL), or both for 48 hours. Transcript levels of (**A**) VE-cadherin and (**B**) α-SMA were measured in RNA extracts using RT-PCR. Protein levels of (**C**) VE-cadherin and (**D**) α-SMA were quantitated by Western blot analysis. (**E**) Immunostaining of VE-cadherin in cells treated with various conditions. (**F**) Endothelial permeability was measured by HRP leakage. Data are expressed as ΔOD470 for permeation of HRP across Transwell filters. In a separate experiment, BPAECs were treated with CHI3L1 (500 ng/mL), exposed to hypoxia (1% oxygen), or both. Transcript levels of (**G**) VE-cadherin and (**H**) α-SMA were measured in RNA extracts using RT-PCR. Protein levels of (**I**) VE-cadherin and (**J**) α-SMA were quantitated by Western blot analysis. (**K**) Immunostaining of VE-cadherin in cells treated with various conditions. (**L**) Endothelial permeability was measured by HRP leakage. Values are mean ± SEM with a minimum of 3 wells in each group. Each experiment was undertaken at least 3 times. Groups were compared by ANOVA with Bonferroni’s post test; follow-up comparisons between groups were conducted using a 2-tailed Student’s *t* test. ****P *≤* 0.0001, ***P *≤* 0.001, **P *≤* 0.01, *P *≤* 0.05 compared with controls.

Loss of VE-cadherin is a hallmark of endothelial injury and loss of endothelial barrier function. Endo-MT is a type of cellular transdifferentiation and has emerged as an important source of α-SMA–expressing, mesenchymal-like cells in obstructive pulmonary vascular lesions in PH. Bovine pulmonary artery endothelial cells (BPAECs) are widely used in PH research under hypoxic conditions, and endo-MT processes have been previously reported (50, 51). To determine if CHI3L1, alone or synergized with TGF-β, acts as a driving force of endothelial barrier loss and endo-MT, we treated these cells with CHI3L1 (500 ng/mL), TGF-β (10 ng/mL), or CHI3L1 (500 ng/mL) and TGF-β (10 ng/mL) combined. We then measured levels of VE-cadherin, an endothelial cell-cell junction protein, and α-SMA, a hallmark of mesenchymal-like cells. Our results showed that treatment with CHI3L1 alone slightly decreased VE-cadherin mRNA levels. The VE-cadherin mRNA levels were significantly lower in the cells treated with TGF-β alone, and its levels were synergistically decreased in the group treated with CHI3L1 and TGF-β combined (Figure 7A). We performed similar analyses to measure levels of α-SMA mRNA in the various treatment groups. While treatment of cells with CHI3L1 alone had no effect on α-SMA, it significantly enhanced the TGF-β–induced increase in α-SMA (Figure 7B). Western blot analyses showed consistent results, with a decrease in VE-cadherin and an increase of α-SMA in cells treated with TGF-β alone and CHI3L1 in combination with TGF-β (Figure 7, C and D). Immunostaining confirmed that combination treatment of TGF-β and CHI3L1 caused the greatest decrease in VE-cadherin expression (Figure 7E) and the greatest amount of α-SMA protein accumulation (Supplemental Figure 4A). Consistently, endothelial permeability measured by HRP leakage was synergistically decreased in the group treated with CHI3L1 and TGF-β or hypoxia combined (Figure 7F).

We next tested the effects of CHI3L1 on endothelial barrier and endo-MT when synergized with hypoxia. Similar approaches were undertaken after treating cells with CHI3L1 (500 ng/mL), hypoxia (1%), or CHI3L1 (500 ng/mL) and hypoxia (1%) combined. In these experiments, CHI3L1 or hypoxia alone did not alter the expression or accumulation of VE-cadherin and α-SMA. The cells treated with CHI3L1 and hypoxia combined demonstrated significant decreases in VE-cadherin transcript and protein levels and significant increases of α-SMA transcript and protein levels (Figure 7, G–J). Immunostaining also demonstrated that CHI3L1 synergizes with hypoxia to promote the loss of VE-cadherin (Figure 7K) and the upregulation of α-SMA (Supplemental Figure 4B). Consistently, endothelial permeability measured by HRP leakage was synergistically decreased in the group treated with CHI3L1 and hypoxia combined (Figure 7L). In combination, these results demonstrate that, when synergized with TGF-β or hypoxia, CHI3L1 is a major driver of endothelial permeability and endo-MT.

### Endothelial cell–specific overexpression of CHI3L1 leads to spontaneous pulmonary vascular remodeling in vivo.

**Figure 8:**
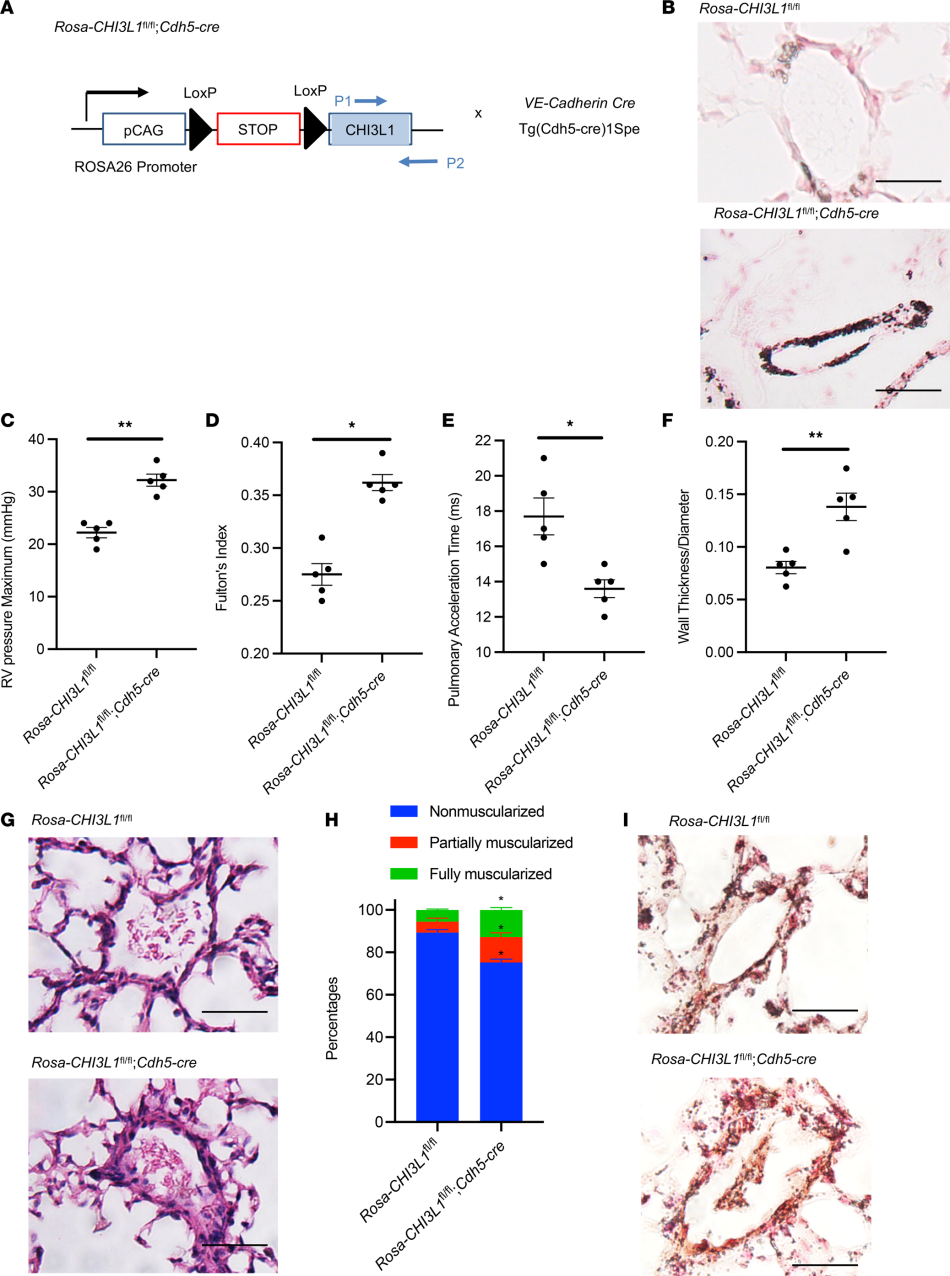
Endothelial cell–specific overexpression of CHI3L1 leads to spontaneous pulmonary vascular remodeling in vivo. (**A**) *Rosa26-CHI3L1*^fl/fl^ mice were crossed with *VE-Cadherin-Cre* mice to generate a mouse model with endothelial cell–specific OE of CHI3L1. (**B**) Endothelial OE of CHI3L1 was shown by IHC. (**C**) RVSP was measured by right heart catheterization. (**D**) Fulton’s index was calculated based on the dry weight of right ventricle and left ventricle plus the septum. (**E**) Echocardiography was used to calculate the PAT. (**F**) Aperio digital pathology slide scanner was used to assess the medial remodeling of pulmonary arteries. The thickness of the medial layer was expressed as a fraction of the external diameter of the pulmonary artery. (**G**) H&E staining of the lungs. Scale bar, 50 μm. (**H**) Vessel wall muscularization of distal pulmonary arteriole (<100 μm in diameter) was quantified based on immunostaining of α-SMA. (**I**) α-SMA staining of the lungs. Scale bar, 50 μm. Values are mean ± SEM with 4–6 mice at 8–9 weeks old in each group. Groups were compared by ANOVA with Bonferroni’s posttest; follow-up comparisons between groups were conducted using a 2-tailed Student’s *t* test. *P *≤* 0.05. **P *≤* 0.01. Images are representatives of 4–6 mice in each group.

We generated Rosa26 locus–targeted *CHI3L1* conditional knockin transgenic mice (*Rosa26-loxP-STOP-LoxP-CHI3L1*-Tg; *Rosa-CHI3L1*^fl/fl^) that can be used to induce endothelial cell–specific overexpression (OE) when crossed with cell-specific promoter-driven Cre mice (Figure 8A). We confirmed CHI3L1 upregulation in endothelial cells, but not in alveolar macrophages, in *Rosa-CHI3L1*^fl/fl^ mice when breeding with *VE-Cadherin-Cre* mice (Figure 8B and Supplemental Figure 5A). Interestingly, at 2 months of age, these mice develop spontaneous vascular remodeling processes as shown by increased RV pressure (Figure 8C), increased Fulton’s index (Figure 8D), decreased PAT (Figure 8E), increased pulmonary vessel wall thickness (Figure 8, F and G, and Supplemental Figure 5B), and increased muscularization of distal pulmonary arterioles shown by α-SMA staining (Figure 8, H and I, and Supplemental Figure 5C). These results demonstrate that endothelial OE of CHI3L1 is sufficient to drive pulmonary vascular remodeling and the development of PH.

## Discussion

Previous studies have identified CHI3L1 as an indicator of disease severity and prognosis in patients with idiopathic PAH (21), PAH associated with scleroderma (22), and IPF (19), but the mechanism of CHI3L1 in the pathogenesis of PH has not been investigated to our knowledge. The importance of CHI3L1-induced responses can be seen in the large number of diseases characterized by inflammation and remodeling in which CHI3L1 excess has been documented (52–60). In many of these disorders, CHI3L1 is likely produced as a protective response based on its ability to decrease epithelial cell apoptosis while stimulating fibroproliferative repair (19). Our laboratory has shown that chitinase-like proteins play important roles in regulating inflammation, an important driver of pulmonary vascular remodeling in PH. We have also found that these proteins modulate injury and repair responses in fibrotic lung diseases (19, 25). We therefore hypothesized that CHI3L1 and its signaling pathways contribute to the vascular remodeling responses that occur in PH.

To test this hypothesis, we first examined circulating CHI3L1 levels in our patients with PH. Similar to previous studies, we found that CHI3L1 correlated directly with pulmonary hemodynamics, including mPAP and pulmonary vascular resistance. We also found that the relationship between CHI3L1 levels and mPAP was similar when we restricted the sample to patients with group 1 PAH and group 3 PH combined, as well as group 1 PAH patients alone. When we restricted the sample to patients with group 3 PH only, the sample size (*n* = 9) was underpowered to detect significant differences. Although we cannot analyze cell-specific contributions of CHI3L1 given the limited human samples, we speculate that endothelial cells and/or myeloid cells are major producers of CHI3L1 in circulation.

In order to determine if the association of elevated CHI3L1 levels with higher PAP was due to a pathogenic role of chitinase-like proteins or merely reflected a marker of disease severity, we examined pulmonary hypertensive responses in genetically engineered mice with absent or enhanced expression of CHI3L1.

We examined pulmonary hypertensive responses in 3 different murine models. Two of these models, chronic hypoxia and MCT, have been used extensively as animal models of PAH. The third model, bleomycin inhalation, results in lung fibrosis and pulmonary vascular remodeling similar to what is seen in patients with PH associated with chronic lung disease. In all 3 models, CHI3L1 mRNA levels in whole lung and protein levels in BAL fluid were increased in comparison with control mice without PH. More importantly, pulmonary hypertensive responses as assessed by measurement of RV hypertrophy, vessel wall thickness, and RVSP were substantially lower in *CHI3L1*^–/–^ mice and higher in transgenic mice that overexpress CHI3L1. In the chronic hypoxia model, we also directly measured PAP by echocardiography and demonstrated that changes in RV mass and vascular wall thickness correlated with pulmonary hemodynamics. The attenuated pulmonary hypertensive responses in the CHI3L1-null mice and the exaggerated responses in mice that overexpress CHI3L1 were consistent across all 3 models and strongly implicate CHI3L1 in the pathogenesis of PH.

In addition to examining the effect of altered CHI3L1 expression on the development of PH, we sought to explore cellular mechanisms by which chitinase-like proteins can modulate pulmonary vascular remodeling. Because CHI3L1 lacks enzymatic activity, how it induces the biological effects described above is unclear. In previous studies, our lab sought to identify receptor-mediated effector functions. Using Yeast Two Hybrid screening and other binding and cellular approaches, we found that CHI3L1 binds to, signals through, and confers tissue responses via IL-13Rα2 and CRTH2 (24, 25). We also demonstrated that CHI3L1 mediates diverse effector responses via different receptors, with IL-13Rα2 (and its co-receptor TMEM219) playing major roles in the inhibition of apoptosis and CRTH2 driving fibroproliferative repair (23–25). In the present study, we profiled the expression of these CHI3L1 receptors in pulmonary vascular endothelial and smooth muscle cells. We found that pulmonary VSMCs express IL-13Rα2, Gal-3, and CRTH2 and endothelial cells express high levels of IL-13Rα2 (and its co-receptor TMEM219).

VSMCs contribute significantly to pulmonary vascular remodeling. Under normal conditions, pulmonary vascular smooth muscle is quiescent, expressing a differentiated contractile phenotype to maintain vascular tone (61). However, in PH, smooth muscle cells can switch to a “synthetic” phenotype in which they secrete inflammatory cytokines, proliferate, and migrate, leading to medial thickening and muscularization of distal normally nonmuscularized vessels (62, 63). Interestingly, it is thought that the α-SMA–positive cells that accumulate in vascular lesions are derived not only from the expansion of resident smooth muscle cells but also from the transition of endothelial cells to a mesenchymal phenotype (endo-MT) (64, 65). In the present study, rCHI3L1 was just as effective as TGF-β and hypoxia in promoting pulmonary vascular smooth muscle proliferation in vitro and synergized with TGF-β and hypoxia to promote endo-MT. The ability of rCHI3L1 to induce smooth muscle cell proliferation was partially blocked by inhibition of CRTH2 and attenuated in smooth muscle cells lacking CRTH2 expression, suggesting that the proliferative effect of CHI3L1 on pulmonary vascular smooth muscle is mediated in part by this receptor.

Endothelial cell apoptosis/dysfunction followed by the emergence of apoptosis-resistant endothelial cells is also believed to play a crucial role in PAH pathogenesis (66–68). Apoptosis-resistant endothelial cells contribute to the obliterative vasculopathy of PAH by inducing endothelial hyperplasia, by thickening of the blood vessel intima, and through vessel occlusion and the development of angio-proliferative plexiform-like lesions, the pathological sine qua non of PAH (69–73). Because bleomycin had the most profound effect to induce the expression of CHI3L1 and IL-13Rα2 in endothelial cells (Figure 6, A and B), we chose bleomycin to challenge the endothelial cells to induce cell death. We found that CHI3L1 was able to inhibit pulmonary arterial endothelial cell death induced by bleomycin and that this effect was mediated in part by the IL-13Rα2/TMEM219 receptor complex, suggesting that CHI3L1 may contribute to the phenotype of these apoptosis-resistant endothelial cells. A previous study by Tan et al. showed that CHI3L1, targeted by microRNA-30a-5p, regulated proliferation and apoptosis of HPAECs under hypoxia (74). Interestingly, consistent with the literature (75, 76), we found that these cells were very resistant to hypoxic stress-induced cell death response. The relevance of CHI3L1-induced apoptosis resistance to induction of PH was further demonstrated in vivo, where endothelial cell–specific OE of CHI3L1 was sufficient to induce spontaneous PH in mice. Together, our findings suggest that the mechanisms by which CHI3L1 contributes to the development of PH are inhibition of endothelial apoptosis, stimulation of VSMC proliferation, and facilitation of endothelial barrier permeability and endo-MT. Interestingly, many of these effects were found to be additive to those of TGF-β and hypoxia, 2 well-known inducers of pulmonary vascular remodeling.

PH often occurs in patients with advanced pulmonary fibrosis and is associated with increased morbidity and mortality. The cause of PH associated with pulmonary fibrosis is unclear. Although hypoxic pulmonary vasoconstriction and fibroproliferative scarring likely contribute to pulmonary vascular remodeling, it is becoming apparent that other mechanisms are also involved. In order to advance the treatment of PH in pulmonary fibrosis, a better understanding of these mechanisms is needed. Previously, we demonstrated that CHI3L1 levels are elevated in IPF and that high levels of CHI3L1 are associated with disease progression in an ambulatory IPF population (19, 25). To investigate the role of CHI3L1 in PH associated with pulmonary fibrosis, we examined the effect of CHI3L1 expression on PH responses in the bleomycin mouse model of pulmonary fibrosis. Bleomycin has been used extensively in previous studies to reveal important mechanisms that promote vascular remodeling in association with lung fibrosis, including endothelin-1 (36), natriuretic peptide receptor–A (37), ACE2 (38, 39), Rho-kinase/ROCK (40), hypoxia signaling (41), adenosine signaling (42, 43), hyaluronan production (45–47), and a population of CXCR2^+^ myeloid-derived suppressor cells (48). As seen with the other models of PH, we found that disrupted expression of CH3L1 abrogated PH in bleomycin-treated mice while OE of CHI3L1 worsened it. Interestingly, the severity of pulmonary fibrosis in CHI3L1-null mice treated with bleomycin was not reduced compared with WT mice, suggesting that lower severity of PH observed in these mice was not the result of a reduction in lung fibrosis. These findings support a major role of CHI3L1 and its receptors in mediating PH responses associated with pulmonary fibrosis.

The consistency of the pulmonary vascular effects of CHI3L1 in our in vitro studies and across multiple animal models of PH strongly suggest that elevated levels of circulating CHI3L1 reported in multiple types of pulmonary hypertensive diseases contribute to pulmonary vascular remodeling and are not merely a biomarker of disease severity. Our results suggest that CHI3L1, a profibrotic molecule, may also have important modulatory roles in endothelial cell apoptosis, smooth muscle cell proliferation, and endo-MT that contribute to the development of PH. Thus, chitinase-like proteins may represent a target for developing new therapies for the treatment of patients with PAH and with PH associated with interstitial lung diseases.

## Methods

### Human patients.

Patients were enrolled as part of a research registry and biorepository from the Rhode Island Hospital Pulmonary Hypertension Center (Institutional Review Board 021911) (77, 78). We approached patients referred to the center for unexplained dyspnea PH evaluation. Blood was drawn at enrollment into the registry, and clinical data were collected as close as possible to the timing of blood draw. Patients were clinically phenotyped and assigned to a WSPH group or to a diseased control group (no PH determined by echocardiogram or hemodynamics using American Society of Echocardiography and hemodynamic criteria) (4, 5, 79) while blinded to CHI3L1 levels. CHI3L1 levels and hemodynamic parameters (mPAP, pulmonary vascular resistance) were natural log–transformed for analyses and were investigated using generalized linear modeling assuming a lognormal distribution. All analyses were conducted in SAS using the GLIMMIX procedure. Statistical significance was designated as *P* < 0.05.

### CHI3L1 ELISA.

Measurement of CHI3L1 was performed with the commercially available ELISA kit (Quantikine, R&D Systems, catalog DC3L10). The minimum detectable dose (MDD) of human CHI3L1 ranged from 1.25 to 8.15 pg/mL. The mean MDD was 3.55 pg/mL. Typical patient serum samples in healthy individuals can be detected in the range of 15.9 to 93.5 ng/mL. The experimental protocol followed Quantikine instructions except for the dilution of serum samples. A 1:500 dilution was used instead of a 1:50 dilution. The standard curve was created by GraphPad (GraphPad Software Inc.) using 4-parameter logistic curve fit. Serum CHI3L1 levels were measured in duplicate.

### Animal models of PH.

Adult male and female 8-week-old C57BL/6 mice (4–7 mice/group/experiment) from The Jackson Laboratory or generated in our facility were used for the preclinical models of PH. Both male and female mice in equal numbers were used in control and experimental groups. In the hypoxia model, mice were subjected to 4 weeks of normoxia or hypoxia (10% oxygen). In the MCT model, mice were challenged with MCT (600 mg/kg weekly for 4 weeks) or vehicle (saline) and sacrificed 1 week after the last MCT injection. In the bleomycin model, mice were given a single intratracheal bleomycin injection (1.25 U/kg) from Teva Parenteral Medicines. Mice were then sacrificed and evaluated 14 days after administration. Fulton’s index was calculated by dissecting the heart to separate the RV free wall from the left ventricle (LV) and septum (S), allowing an assessment of RV hypertrophy by the weight ratio of RV/(LV + S). The lungs were harvested, and Aperio digital pathology slide scanner was used to assess the medial remodeling of pulmonary arteries. The thickness of the medial layer was expressed as a fraction of the external diameter of the pulmonary artery. To determine muscularization of distal pulmonary arteriole vessel wall, α-SMA immunostaining was performed (Abcam ab5694). Each vessel was categorized as being nonmuscular, partially muscularized (muscularization less than 3/4 of the vessel circumference), or fully muscularized (muscularization in more than 3/4 of vessel circumference). The percentage muscularization of pulmonary vessels was determined by dividing the number of vessels in each category by the total number counted for that experimental group. Pulmonary vessels 100 μm in diameter or fully muscularized vessels associated with bronchi were excluded. All mice were congenic on a C57BL/6 background and were genotyped as previously described. *WT* mice were purchased from The Jackson Laboratory. Genetically modified mice that were used in the study include a) *CHI3L1*-null mutant mice (*CHI3L1*^–/–^); b) doxycycline-inducible, cc10 promoter–driven, lung-specific, human *CHI3L1*–overexpressing, transgenic (*CHI3L1*-Tg) mice; and c) *Rosa26* locus–targeted, *CHI3L1* conditional knockin transgenic mice (*Rosa26-loxP-STOP-LoxP-CHI3L1* Tg; *Rosa-CHI3L1*^fl/fl^) that could be used to induce cell-specific CHI3L1 OE when crossed with cell-specific promoter-driven Cre mice. All 3 strains were generated in our facility. The *Rosa-CHI3L1*^fl/fl^ mice were crossed with mice in which Cre was expressed using endothelial cell–specific (*VE-cadherin*) promoter [Tg(*Cdh5-cre*)1Spe, The Jackson Laboratory] to define the roles of CHI3L1 in endothelial cells. These mice tend to develop severe acanthosis and hyperkeratosis of the skin. Soft gel diet was provided to these mice to make sure they received enough nourishment to survive until week 6. Experiments were conducted in the surviving mice.

### RVSP measurements.

In vivo hemodynamic measurements were performed using Millar catheters to measure RV pressure through the jugular vein and left ventricular pressure through the carotid artery. Animals were then euthanized for tissue harvest via exsanguination under anesthesia.

### Echocardiography.

To measure PAT in adult mice anesthetized with isoflurane, echocardiography was performed using a VisualSonics Vevo 2100 High-Resolution Imaging System to assess cardiac structure and function. Once each mouse was adequately sedated, the chest was shaved, and the animal was placed in a supine position. The animal was maintained normo-thermic with a heating lamp. Ultrasonic gel was liberally applied to the chest wall, prior to imaging, to optimize cardiac images. Following echocardiographic examinations, mice were watched until freely moving, grooming, and eating.

### Quantification of lung collagen.

The collagen content was determined by quantifying total soluble collagen using the Sircol Collagen Assay kit (Biocolor, Accurate Chemical and Scientific Corp, S1000) per manufacturer’s instructions.

### Gene expression analysis.

Cells processed from human blood were lysed in TRIzol reagents Thermo Fisher Scientific), and total cellular RNA was extracted by QIAGEN RNeasy kit per manufacturer’s instructions. From the mRNA, cDNA was synthesized using the Bio-Rad iScript cDNA Synthesis Kit per manufacturer’s instructions. The corresponding mRNA level was then measured using RT-PCR. The primer sequences were obtained from PrimerBank of Massachusetts General Hospital or were the same as ones previously used.

### Cell culture.

HPASMCs were obtained from Lonza. Cells were treated with CHI3L1 (500 ng/mL), TGF-β (10 ng/mL), or both for 48 hours. In a separate experiment, cells were treated with CHI3L1 (500 ng/mL), exposed to hypoxia (1% oxygen in hypoxia incubator chamber, Stemcell Technologies 27310), or both. BrdU incorporation ELISA was used to quantitate smooth muscle cell proliferation according to manufacturer-provided protocols (Abcam ab126556). Briefly, cells were incubated in various conditions (CHI3L1, CRTH2 inhibitor CAY10471, or DMSO as vehicle control) for 24 hours, followed by treatment with 10 μM BrdU for 1 hour. In addition, WST-1 Cell Proliferation Assay was used to quantitate smooth muscle cell number according to manufacturer-provided protocols (Roche 05015944001). HPAECs were obtained from Lonza. TUNEL staining was performed and TUNEL-positive cells were counted to evaluate cell death. Endothelial cell apoptosis was further evaluated with APC–annexin V apoptosis detection kit with PI (BioLegend 640932) according to the manufacturer’s recommended protocol. Briefly, cells were treated with various conditions, then stained with annexin V–APC for 15 minutes at 37°C in Annexin V Binding Buffer. PI (0.03 μg/test catalog 421301) was added 5 minutes prior to FACS analysis. For endo-MT experiments, BPAECs were obtained from VEC Technologies. Cells were cultured in T75 flasks using MCDB 131 medium (Thermo Fisher Scientific) at 37°C in 5% CO_2_. Cells were subcultured approximately every 2 days by aspirating off medium, washing once with PBS, detaching cells using 5 mL of trypsin, and neutralizing with 15 mL of fresh medium. Cells were used within 11 passages. Cells were seeded on 6-well plates for RNA and protein extraction and 4-well chamber slides for immunofluorescence. Cells were then treated with CHI3L1 (500 ng/mL), TGF-β (10 ng/mL), CHI3L1 (500 ng/mL) and TGF-β (10 ng/mL) together, or neither (control treatment) and incubated at 37°C for 48 hours. In separate experiments, cells were treated with CHI3L1 (500 ng/mL), hypoxia (1%), CHI3L1 (500 ng/mL) and hypoxia (1%) together, or neither and incubated at 37°C for 48 hours. Cells were collected and levels of VE-cadherin and α-SMA were analyzed using RT-PCR, Western blot analysis, and immunostaining. Specifically, BPAECs were lysed with 0.2% Triton X-100 in PBS for 20 minutes. Total cellular RNA was extracted by QIAGEN RNeasy kit per manufacturer’s instructions. cDNA was synthesized from mRNA using the Bio-Rad iScript cDNA Synthesis Kit. Levels of VE-cadherin and α-SMA mRNA transcript were then measured using RT-PCR. For Western blot analysis, proteins from lysed BPAECs were separated using SDS-PAGE, and membrane transfer was performed using the Bio-Rad Trans-Blot Turbo Transfer System. Membranes were then blocked for 1 hour using 5% milk in TBS-Tween (TBST). Primary antibodies for VE-cadherin (Abcam, ab119339), α-SMA (Abcam, ab5094), and tubulin (Thermo Fisher Scientific, 62204) were diluted with 5% BSA in TBST in a 1:500 ratio and added to respective membranes. After incubating overnight, membranes were washed with PBS 3 times and then incubated for 1 hour with anti-rabbit (diluted 1:3,000 with 5% milk in TBST) (Thermo Fisher Scientific, 31460) and anti-mouse (diluted 1:2,000 with 5% milk in TBST) (Thermo Fisher Scientific, 31430) secondary antibodies, respectively. Membranes were then washed 3 times with PBS and incubated for 5 minutes with a 1:1 mixture of Thermo Fisher Scientific SuperSignal West Femto Maximum Sensitivity Substrate to Enhancer Solution. Bio-Rad’s protein blotting detection and imaging machine was used to visualize the membranes and detect protein levels. Immunostaining was used to visualize changes in levels of VE-cadherin and α-SMA in the various treatment groups. Cells were fixed on 4-chamber Chamber slides (Thermo Fisher Scientific, 154461PK) with 100% methanol (–20°C) at room temperature for 5 minutes. Normal goat serum (1% in PBS) was added as a blocking buffer to each chamber and incubated for 1 hour. Each chamber then received 200 μL of either anti–VE-cadherin primary antibody from a rabbit (Abcam, ab331687) diluted at 1:100 or anti–α-SMA primary antibody from a mouse (Abcam, ab7817) diluted at 1:200 and incubated overnight at 4°C. The cells were then washed with PBS 3 times for 10 minutes and incubated for 1 hour at room temperature in the dark with Alexa Fluor 488–labeled anti-rabbit (Thermo Fisher Scientific, A26034) or Alexa Fluor 594–labeled anti-mouse (Thermo Fisher Scientific, A11005) secondary antibodies, respectively. DAPI staining was performed for each treatment group to confirm the presence of cells. Fluorescence microscopy was then used to analyze cells. For HRP permeability assay, cells were cultured using 8.0 μm pore size polycarbonate cell culture inserts that fit in a 24-well cluster cell culture plate. A total of 0.15 μM HRP was added to the upper chamber. Data are expressed as ΔOD470 for permeation of HRP across Transwell filters.

### Statistics.

Mouse data are expressed as mean ± SEM. As appropriate, groups were compared by 2-way ANOVA with Bonferroni’s posttest; follow-up comparisons between groups were conducted using a 2-tailed Student’s *t* test. A *P* ≤ 0.05 was considered significant. In human studies, parametric data were compared by 2-tailed Student’s *t* test. Nonparametric data were compared using the Mann-Whitney *U* test. Statistical analysis was performed using GraphPad (GraphPad Software Inc.). Graphs were generated using Microsoft Excel and GraphPad.

### Study approval.

Animal experiments were approved by the Institutional Animal Care and Use Committee of Brown University in accordance with federal guidelines. Human studies were approved by the Rhode Island Hospital Institutional Review Board (Providence, Rhode Island, USA).

## Author contributions

JRK and YZ were responsible for conception and design; XS, EN, CN, PS, AXY, DY, CEV, JB, AV, JA, DB, MP, GB, QL, EOH, SR, CGL, HY, GC, and JRK were responsible for analysis and interpretation; and CEV, JRK, and YZ were responsible for drafting the manuscript for important intellectual content.

## Supplementary Material

Supplemental data

## Figures and Tables

**Table 1:**
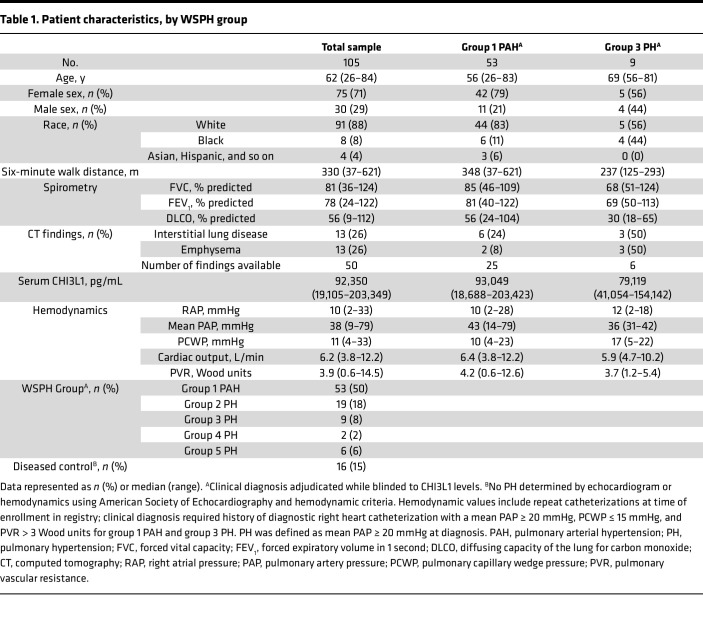
Patient characteristics, by WSPH group

## Version 1 08/11/2022

In-Press Preview

## Version 2 09/22/2022

Electronic publication
