# Supplementary material for: Chitinase 3 like 1 contributes to the development of pulmonary vascular remodeling in pulmonary hypertension
Source: JCI Insight. 2022 Sep 22;7(18):e159578. doi: 10.1172/jci.insight.159578 (PMC9675485; doi:10.1172/jci.insight.159578)
Supplement: Supplemental data [file jciinsight-7-159578-s046.pdf]

Supplementary Fig 1.

A

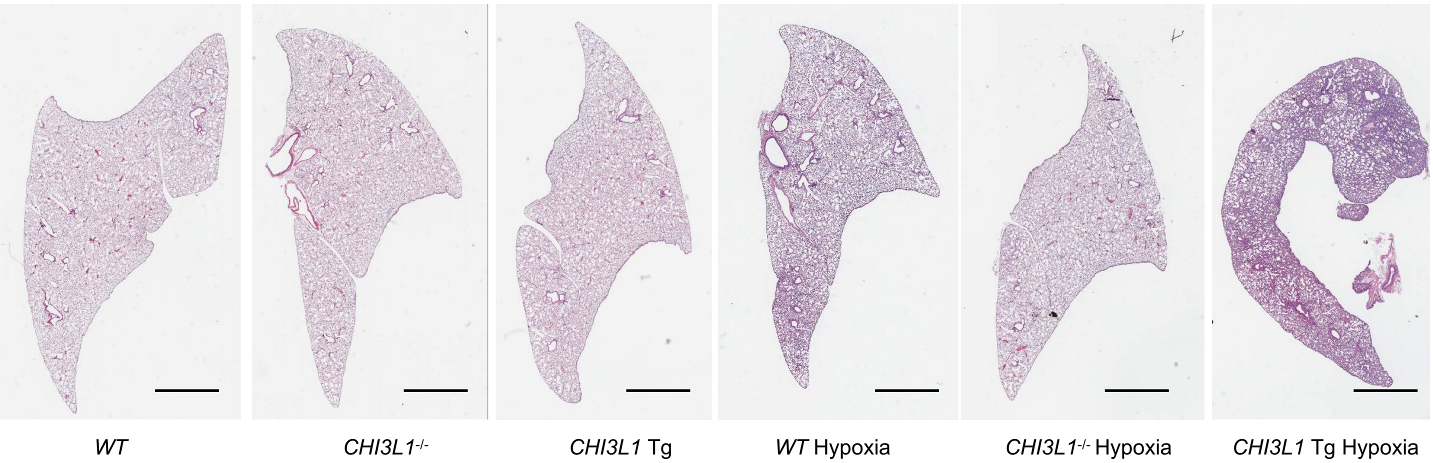

B

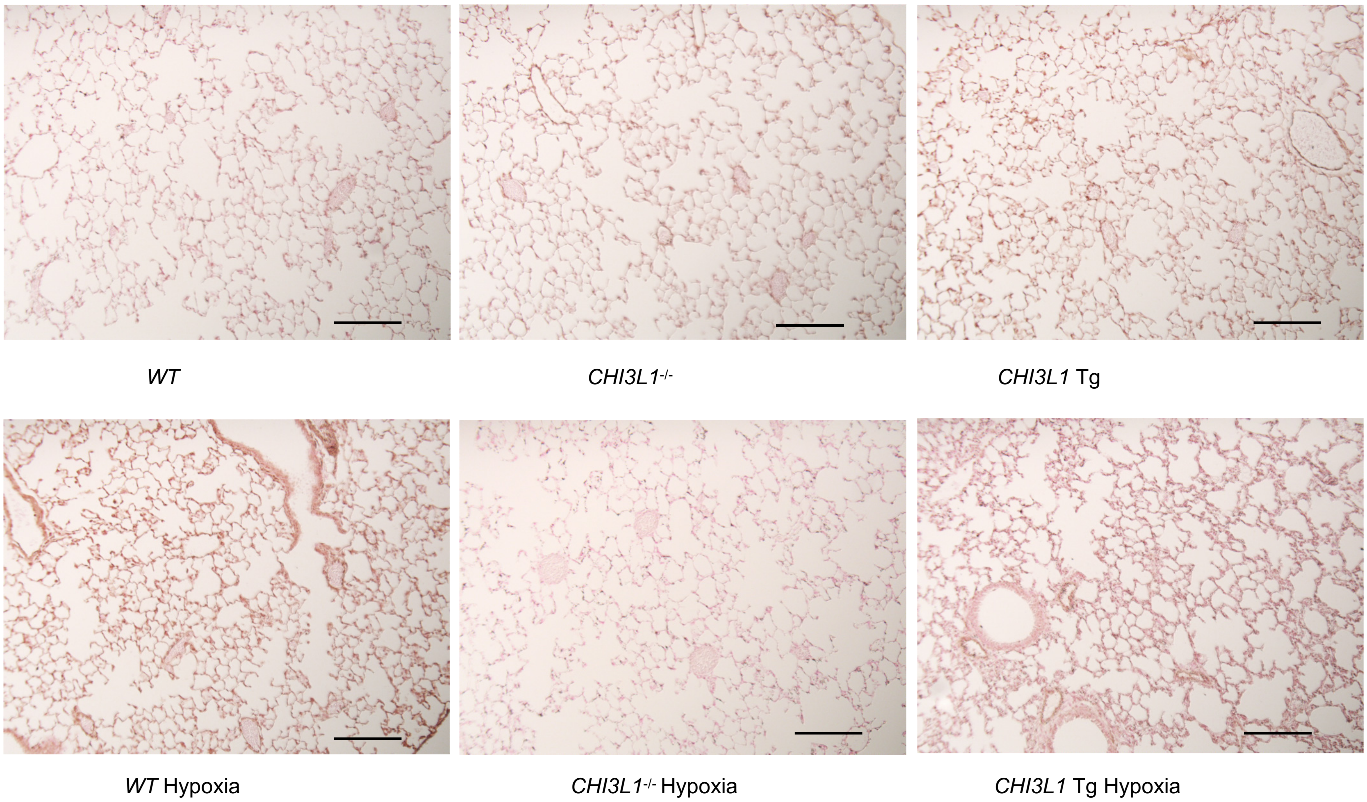

Supplementary Figure 1. CHI3L1 plays a critical role in vascular remodeling responses in hypoxia-induced pulmonary hypertension model. *WT* (+/+), *CHI3L1* null mice (-/-), and *CHI3L1* transgenic overexpression mice (Tg+) were subjected to 6 weeks of normoxia or hypoxia (10% Oxygen). (A) H&E staining of the lungs from *WT* mice, *CHI3L1* null mice, and *CHI3L1* Tg mice exposed to normoxia or hypoxia. Scale bar, 2mm. (B) α-SMA staining of the lungs from *WT* mice, *CHI3L1* null mice, and *CHI3L1* Tg mice exposed to normoxia or hypoxia. Scale bar, 200μm. Images are representatives of 4-6 mice in each group.

Supplementary Fig 2.

A

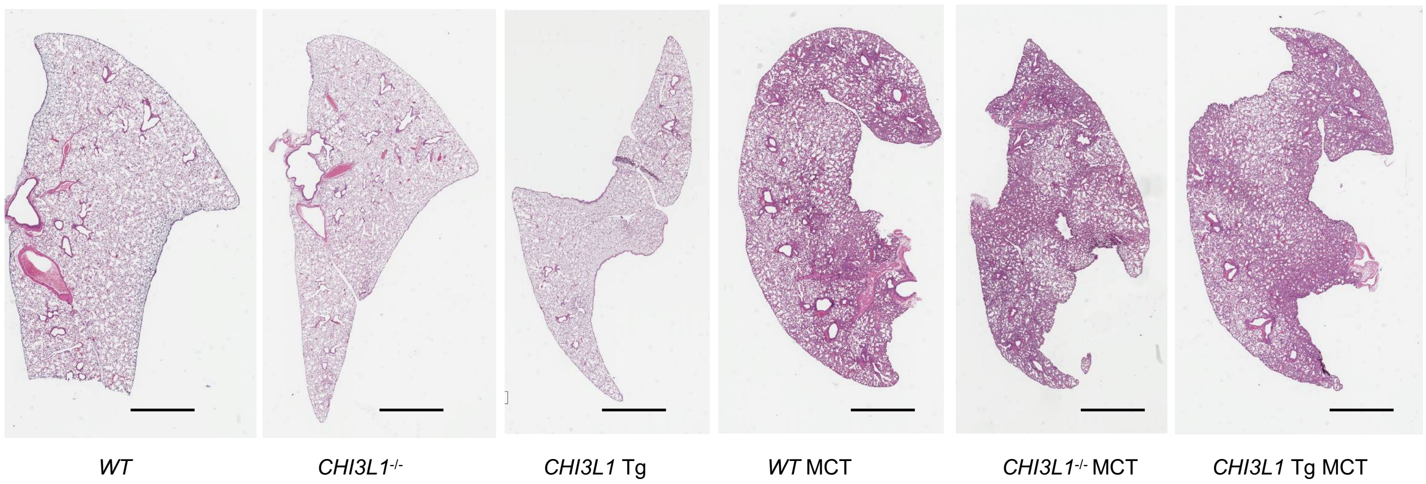

B

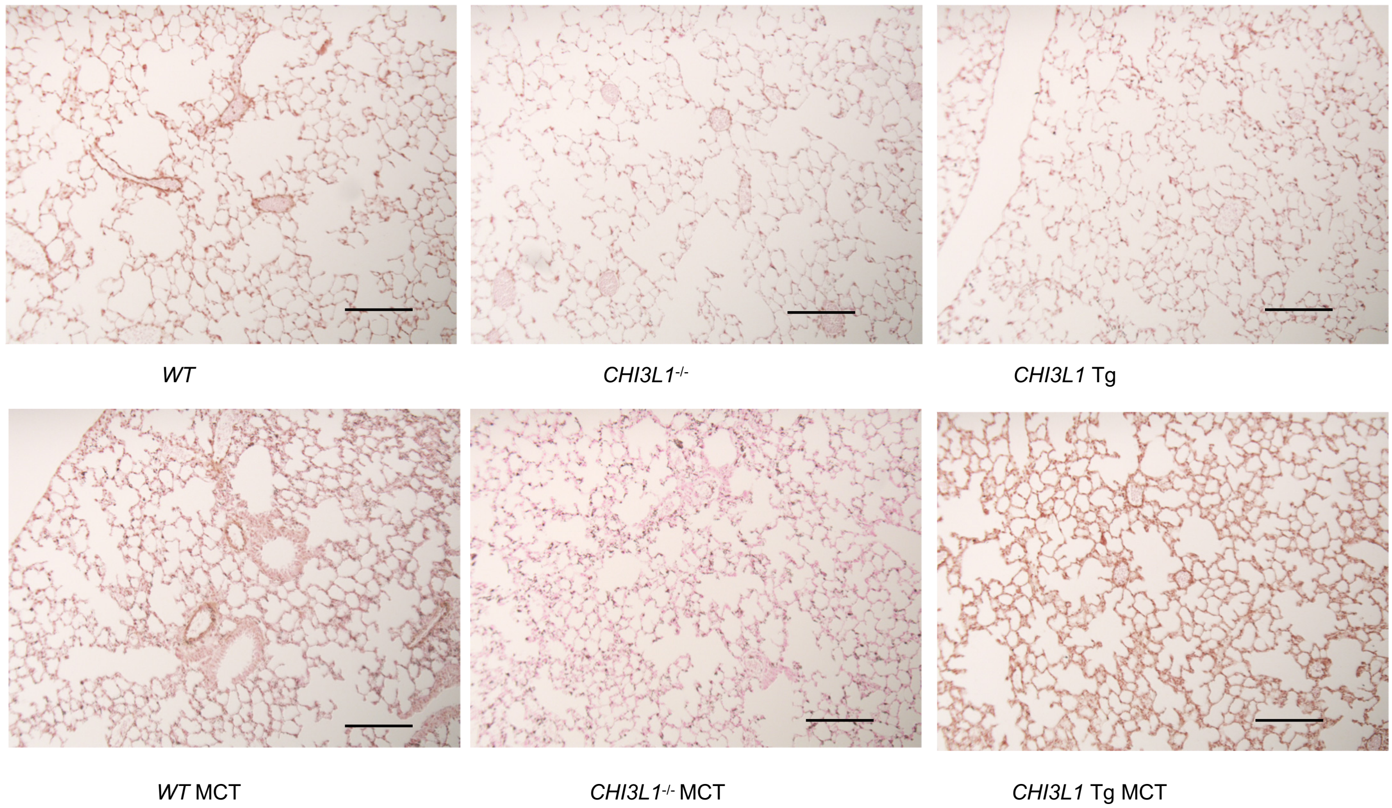

Supplementary Figure 2. CHI3L1 plays a critical role in vascular remodeling responses in MCT-induced pulmonary hypertension model. *WT* (+/+), *CHI3L1* null mice (-/-), and *CHI3L1* transgenic overexpression mice (Tg+) were challenged with monocrotalin (MCT) (600mg/kg weekly for four weeks) or vehicle and sacrificed one week after the last MCT injection. (A) H&E staining of the lungs from *WT* mice, *CHI3L1* null mice, and *CHI3L1* Tg mice. Scale bar, 2mm. (B) α-SMA staining of the lungs from *WT* mice, *CHI3L1* null mice, and *CHI3L1* Tg mice. Scale bar, 200μm. Images are representatives of 4-7 mice in each group.

Supplementary Fig 3.

A

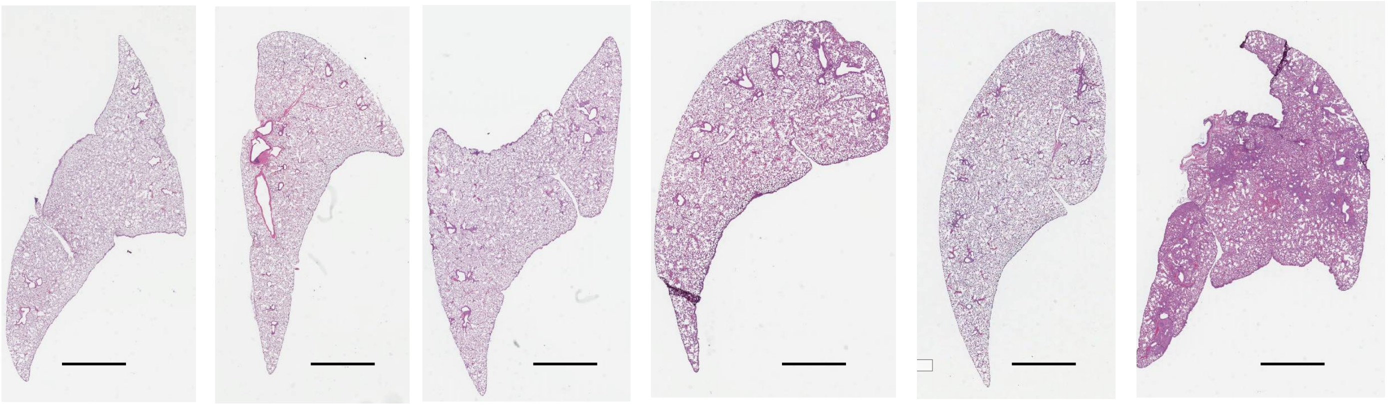

WT | *CHI3L1*$^{-/-}$ | *CHI3L1* Tg | *WT* Bleo | *CHI3L1*$^{-/-}$ Bleo | *CHI3L1* Tg Bleo

B

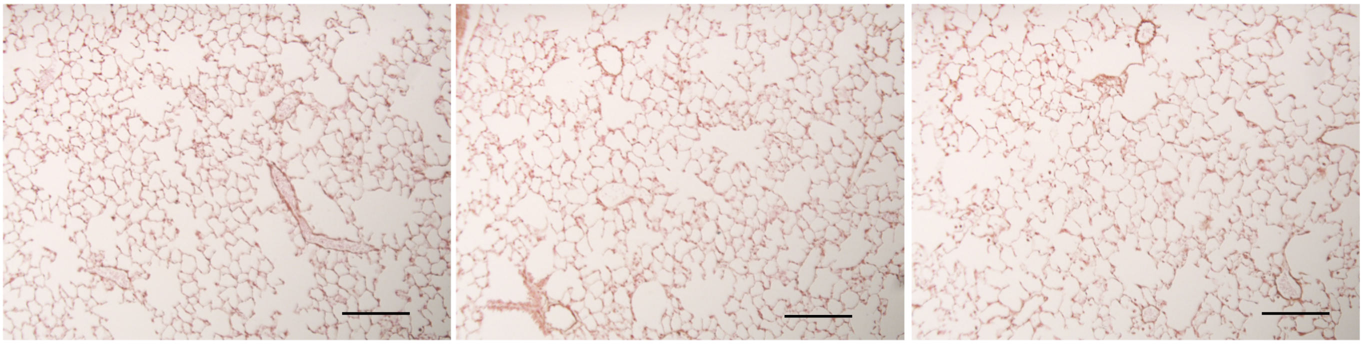

WT | *CHI3L1*$^{-/-}$ | *CHI3L1* Tg

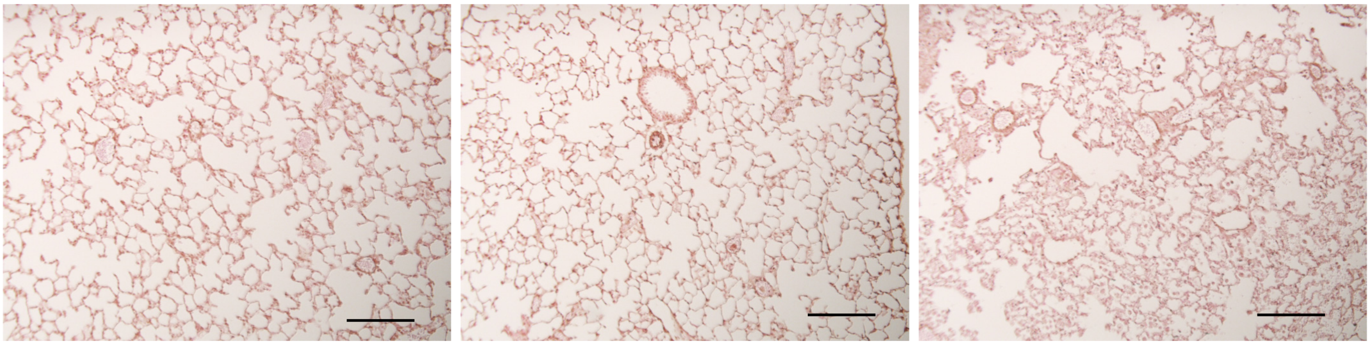

WT Bleo | *CHI3L1*$^{-/-}$ Bleo | *CHI3L1* Tg Bleo

Supplementary Figure 3. CHI3L1 plays a critical role in vascular remodeling responses in bleomycin-induced pulmonary hypertension model. *WT* (+/+), *CHI3L1* null mice (-/-), and *CHI3L1* transgenic overexpression mice (Tg+) were subjected to intratracheal PBS or bleomycin administration. Mice were sacrificed at Day 14 after bleomycin challenge . (A) H&E staining of the lungs from *WT* mice, *CHI3L1* null mice, and *CHI3L1* Tg mice. Scale bar, 2mm. (B) α-SMA staining of the lungs from *WT* mice, *CHI3L1* null mice, and *CHI3L1* Tg mice. Scale bar, 200μm. Images are representatives of 4-6 mice in each group.

Supplementary Fig 4.

A

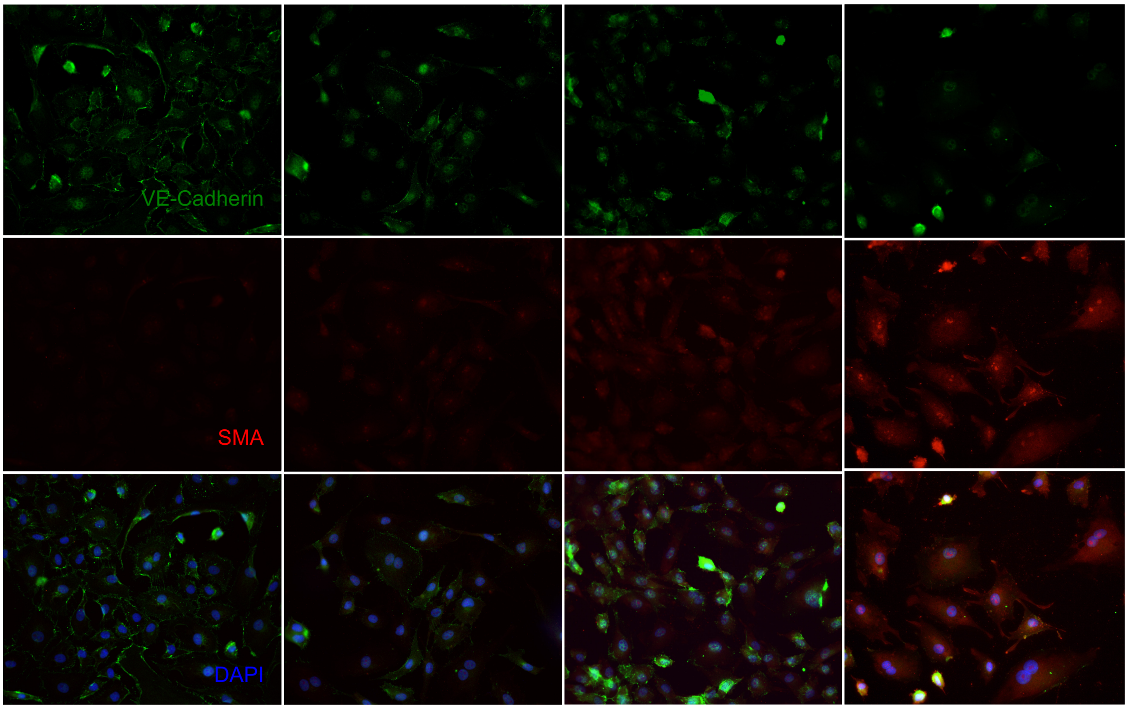

B

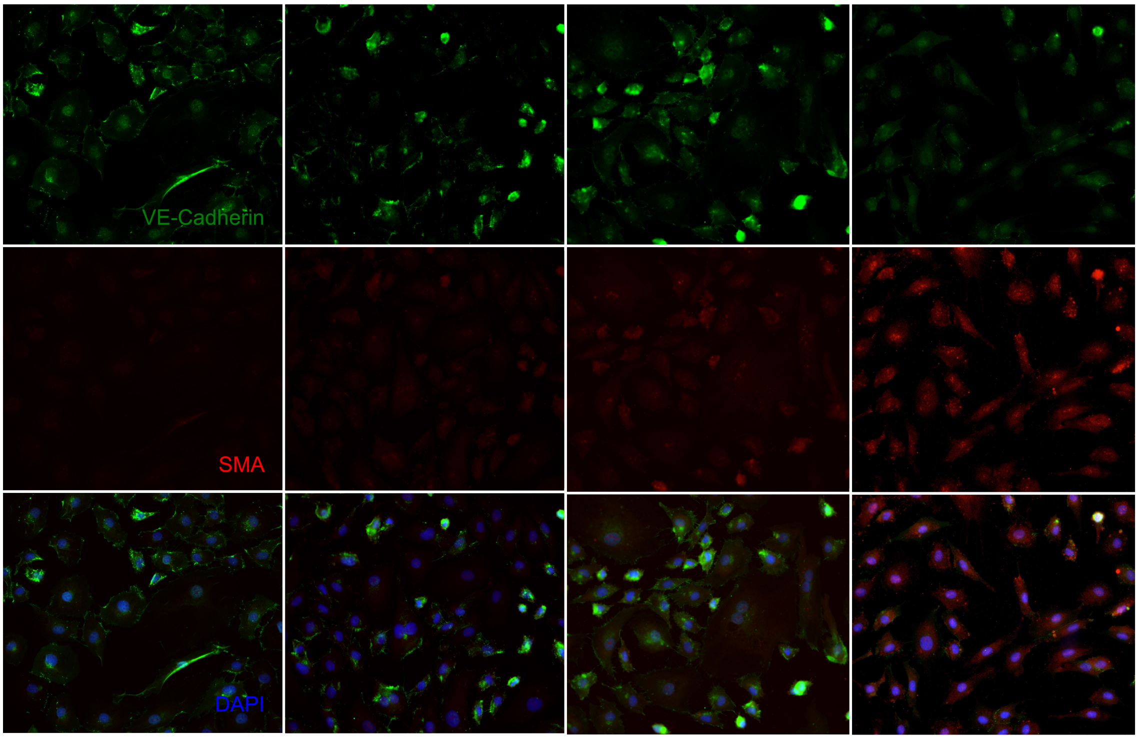

Supplementary Figure 4. CHI3L1 synergizes with TGF-β or hypoxia, and promotes endothelial-to-mesenchymal transition. (A) Bovine pulmonary arterial endothelial cells were treated with CHI3L1 (500ng/ml), TGF-β (10ng/ml), or together for 48 hours. (B) In a separate experiment, bovine pulmonary arterial endothelial cells were treated with CHI3L1 (500ng/ml), exposed to hypoxia (1% Oxygen), or both. Immunostaining of VE-Cadherin and α-SMA in cells treated with various conditions. Each experiment was undertaken at least 3 times.

Supplementary Fig 5.

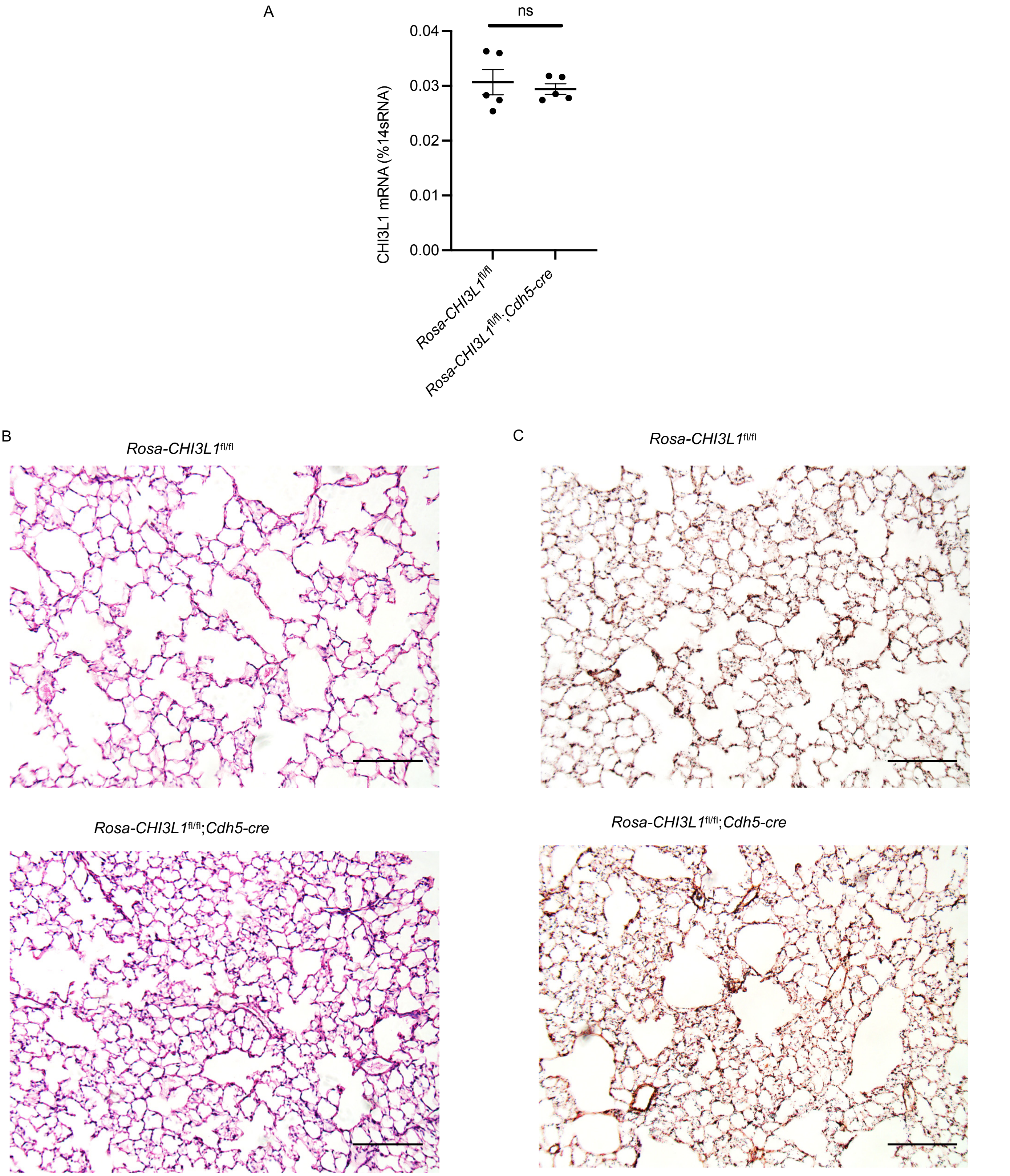

Supplementary Figure 5. Endothelial-specific overexpression of CHI3L1 leads to spontaneous pulmonary vascular remodeling *in vivo*. *Rosa26-CHI3L1*$^{fl/fl}$ mice were crossed with *VE-Cadherin-Cre* mice to generate a mouse model with endothelial-specific overexpression of CHI3L1. (A) Expression of CHI3L1 was examined in alveolar macrophages. (B) H&E staining of the lungs. (C) α-SMA staining of the lungs. Values are mean ± SEM with 4-6 mice at 8-9 weeks old in each group. Groups were compared by ANOVA with Bonferroni's post test; follow-up comparisons between groups were conducted using a two-tailed Student's t-test. Images are representatives of 4-6 mice in each group. ns, not significant. Scale bar, 200μm.
